# Modeling Differential Enthalpy of Absorption of CO_2_ with Piperazine as a Function of Temperature

**DOI:** 10.1021/acs.jpcb.1c10755

**Published:** 2022-02-28

**Authors:** Mayuri Gupta, Eirik Falck da Silva, Hallvard F. Svendsen

**Affiliations:** † Department of Chemical Engineering, Norwegian University of Science and Technology, Sem Sælands vei 4, Trondheim 7491, Norway; ‡ Department of Process Technology, SINTEF Industry, Trondheim 7034, Norway

## Abstract

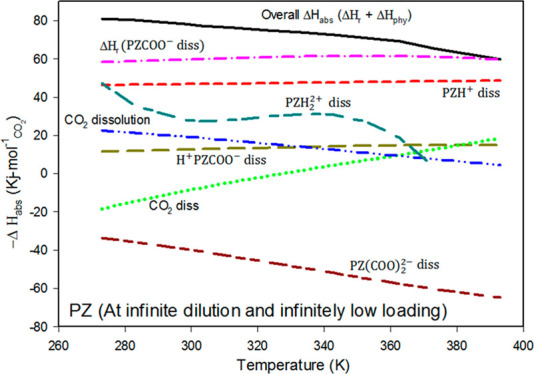

Temperature-dependent correlations for equilibrium constants (ln *K*) and heat of absorption (Δ*H*_abs_) of different reactions (i.e., deprotonation, double deprotonation, carbamate formation, protonated carbamate formation, dicarbamate formation) involved in the piperazine (PZ)/CO_2_/H_2_O system have been calculated using computational chemistry based ln *K* values input to the Gibbs–Helmholtz equation. This work also presents an extensive study of gaseous phase free energy and enthalpy for different reactions using composite (G3MP2B3, G3MP2, CBS-QB3, and G4MP2) and density functional theory [B3LYP/6-311++G(d,p)] methods. The explicit solvation shell (ESS) model and SM8T solvation free energy coupled with gaseous phase density functional theory calculations give temperature-dependent reaction equilibrium constants for different reactions. Calculated individual and overall reaction equilibrium constants and enthalpies of different reactions involved in CO_2_ absorption in piperazine solution are compared against experimental data, where available, in the temperature range 273.15–373 K. Postcombustion CO_2_ capture (PCC) is a temperature swing absorption–desorption process. The enthalpy of the solution directly correlates with the steam requirement of the amine regeneration step. Temperature-dependent correlations for ln *K* and Δ*H*_abs_ calculated using computational chemistry tools can help evaluate potential PCC solvents’ thermodynamics and cost-efficiency. These correlations can also be employed in thermodynamic models (e.g., e-UNIQUAC, e-NRTL) to better understand postcombustion CO_2_ capture solvent chemistry.

## 1 Introduction

Postcombustion CO_2_ capture (PCC) by amine scrubbing is the most mature technology used to control global warming and the resulting global climate change.^1−3^ The design and cost of the postcombustion CO_2_ capture plant profoundly depend on the enthalpy of the CO_2_ capture amine solution. The enthalpy of solution directly correlates with the steam requirement of an amine regeneration step in PCC as amine scrubbing relies on the thermal swing solvent regeneration. The steam cost related to solvent regeneration amounts to roughly half of the cost of PCC plant operation. Therefore, accurate estimation of absorption enthalpy is critical in ensuring efficient plant design while minimizing overall costs.^4,5^

Piperazine (PZ), a cyclic diamine with two secondary amine nitrogens and a six-membered saturated ring, is considered to be a promising PCC solvent. PZ is an efficient rate promoter with high absorption capacity (2 mol of CO_2_ per mole of amine) and is resistant to oxidation and thermal degradation.^6−8^ PZ is also used widely in blended amine solutions such as PZ activated MDEA,^9−13^ AMP,^14,15^ MEA,^16,17^ and K_2_CO_3_^18,19^ solutions. In this work, the heat of absorption of CO_2_ in aqueous PZ is estimated by employing the explicit solvation shell model and the SM8T continuum solvation model coupled with quantum mechanical DFT calculations with the help of the Gibbs–Helmholtz equation. Our earlier work introduced the methodology of calculating temperature-dependent enthalpy correlations based on ln *K* value input to the Gibbs–Helmholtz equation, calculated by employing various gaseous and solution phase computational chemistry methods.^20^ Experimental measurement of the enthalpy of absorption of adding CO_2_ to PCC alkanolamines solvents comprises multiple error sources. It, therefore, has a high degree of variability depending on experimental conditions and external factors. In the literature, calorimetric heats of absorption of CO_2_ absorption reaction with few amines and alkanolamines have been reported with different uncertainties.^21^ For example, the enthalpies of CO_2_ absorption in the solutions of monoethanolamine (MEA), diethanolamine (DEA), and *N*-methyldiethanolamine (MDEA) contained uncertainties of ±10% and ±5% as reported by Carson et al.^22^ and Merkley et al.,^23^ respectively. There could be various sources of error in experimental heat of solution measurements, e.g., the fluctuation in the CO_2_ amount in the reactor gas phase, in the calculation of heat released by integration of the heat flux curve, setting of baseline and integration limits, limited range of operating temperatures and pressures, and the ambiguity in the amount of CO_2_ added to the calorimeter from the external cylinder due to temperature fluctuations as a result of the Joule-Thomson effect.^24,25^ The slow kinetics of some alkanolamine–CO_2_ absorption reactions may introduce other errors such as low and scattered enthalpy values.^22^ Calculation of CO_2_ absorption in alkanolamine solutions employing computational chemistry methods provides a valuable tool to estimate temperature effects on the heat of CO_2_ absorption of various PCC solvents within experimental error bars having a minimal cost of calculations (∼ couple of hours on one PC unit).

Various equilibrium reactions involved in the PZ/CO_2_/H_2_O system have been studied in this work. We calculated equilibrium constants and temperature dependencies of the equilibrium reactions involved in CO_2_ absorption using various gaseous and solution phase computational chemistry methods. The comprehensive understanding of the different equilibrium reactions involved is sought, and the corresponding absorption enthalpy of each equilibrium reaction is calculated using the Gibbs–Helmholtz equation. Calculated enthalpies of different equilibrium reactions and overall heats of solution are compared against experimental data for PZ, where available. The individual effect of protonation and carbamate formation reaction enthalpy on the overall heat of solution is also studied. The overall energy demand of a potential PCC solvent can be estimated by employing this methodology.

## 2 Computational Details

Gaseous phase optimization was carried out in Spartan 08,^26^ employing density functional theory (DFT) at the B3LYP level using the 6-311++G (d, p) basis set. The absence of any imaginary frequencies in the minima was confirmed by performing frequency calculations. Gaussian–n theories (G3MP2B3, G3MP2, G4MP2, CBS-QB3) and density functional theories (DFT) at B3LYP/6-311++G (d,p) level were used to calculate total enthalpies and free energies in the gaseous phase. G3MP2B3, G3MP2, CBS-QB3, and DFT thermochemical calculations were done in Gaussian 03;^27^ Gaussian 09 was used for G4MP2 thermochemical calculations.^28^ Aqueous phase geometries were fully optimized using the SM8 solvation model in Spartan 08 at B3LYP/6-311++G (d, p) level of theory. Single point energy calculations on the optimized geometry of the molecule obtained are used to study the solvation effects with SM8T solvation models. Conformer search was performed in both the gaseous and solvent phases. More details on conformer search and lowest energy conformer structures are given in the Supporting Information (Figure S3).

To study the effect of temperature, SM8T calculations^29^ were performed in the temperature range of a typical PCC process, i.e., 273.15–373 K using Gamessplus.^30^ All SM8T calculations were carried out using density functional theory at the SM8T/B3LYYP/6-311++G (d, p)//SM8/B3LYP-6-311++G (d, p) level. The SM8T continuum solvation model is parametrized to study neutral molecules and not parametrized to study the temperature effects of solvation energies of ionic molecules. However, SM8T gives good qualitative results for solvation energies of ions,^29,31^ and also, in our recent work, we have shown that temperature dependency of various equilibrium constants is reproducible within experimental error bars using the SM8T solvation energy values.^3,32−34^ In this work, solvation energies for neutral molecules at 298 K and the temperature dependency of solvation energies of all species involved in the PZ/CO_2_/H_2_O system chemistry are calculated with SM8T. The explicit solvation shell (ESS) model is employed to calculate the solvation free energies of ionic species involved in this work. The cluster of solute and five water molecules were extracted from molecular dynamics simulations of the solute in the bulk solvent. The motivation for employing five explicit water molecules to capture solute and explicit water interactions is explained in detail by da Silva et al.^35^ In the present work, we have used five explicit water molecules as well because of following underlying considerations. First, the cancellation of different common errors introduced from gas phase calculations, entropy calculations, and solvation free energy calculations is apparent by using a fixed number of explicit water molecules as compared to having a varying number of explicit water molecules for different molecules. Second, the studied piperazine molecule species in the present work are small organic molecules, which are fairly solvated by employing five explicit water molecules. Third, having a consistent and reasonable explicit water molecule help in maintaining the computational costs of the calculations within modest limits. Fourth, the results presented by da Silva et al.^35^ on similar amine molecules are encouraging to use the same number of explicit water molecules in the present work. However, in the literature, there is a considerable discussion on the number of explicit water molecules required to accurately capture solute–solvent interactions. An approach of introducing explicit water molecules until the calculated solvation free energies converge is proposed by Bryantsev et al.^71^ This approach could be more reliable compared to methods which are based on size and polarization of ionic molecules. However, on the contrary, this approach is computationally costly and substantial reduction of errors introduced due to variations in gaseous and solvation phase calculations within one studied data set of molecules may not be achieved.

The solute and five explicit water molecule clusters were fully optimized using quantum mechanical calculations as explained by da Silva et al.^35^ The cluster solvation energies are calculated by using the Poisson–Boltzmann-based continuum solvation model in the DivCon code.^36^ The continuum solvation energy computations were single-point calculations on the optimized HF/6-31+G (d) solute-explicit water clusters. The Poisson–Boltzmann model calculations were performed at the AM1 level, as the model is not parametrized at the HF level of theory. A summary of the Poisson–Boltzmann model and further details of molecular dynamics simulations are given in the Supporting Information and previous publications.^37−44^ The Gaussian 03 software was used for quantum mechanical ESS calculations, and all MD simulations were performed using Sander from the AMBER 12 suite.^45^

## 3 Thermodynamic Framework

### 3.1 Physical and Chemical Absorption of CO_2_

The amount of CO_2_ absorbed in a PCC solvent is evaluated by the physical solubility of CO_2_ and the chemical equilibrium of various aqueous phase reactions involved for the amine/CO_2_/H_2_O system. The summation of enthalpies of different reactions occurring between CO_2_, H_2_O, and amine (PZ) in aqueous phase and enthalpy of physical absorption of CO_2_ from gaseous to liquid phase gives the overall enthalpy of reaction of gaseous CO_2_ with aqueous piperazine (PZ), as described by eq 1:
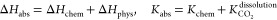
1

### 3.2 Solution-Phase Chemical Equilibrium

Various reactions occurring in the aqueous PZ–CO_2_ system involve the formation of bicarbonate, carbonate, monocarbamate, dicarbamate, and a zwitterion.^46^ The following reactions summarize the chemical absorption of CO_2_ in an aqueous PZ solution.^19,47^

1. Ionization of H_2_O
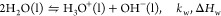
2
2. CO_2_ dissolution
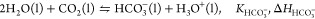
3
3. Bicarbonate ion dissociation
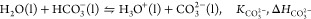
4
4. Diprotonated and protonated piperazine dissociation
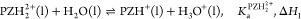
5
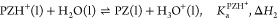
6
5. Piperazine carbamate formation reaction
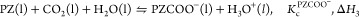
7
6. Protonated piperazine carbamate dissociation
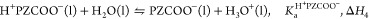
8
7. Piperazine dicarbamate formation reaction
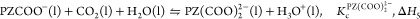
9

At infinite dilution conditions (i.e., at the beginning of the absorption process), the overall reaction of chemical absorption of CO_2_ in PZ (cyclic diamine) can be represented by the following equation^48^
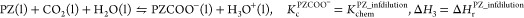
10

The equilibrium constants of the above reactions are calculated from the overall Gibbs free energy
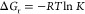
11Employing thermodynamic cycles (given in Supporting Information Figure S1) for different reactions in this work, Δ*G*_r_ is calculated by adding gaseous and aqueous phase Gibbs free energies of the corresponding reactions. *R* is the universal gas constant.

### 3.3 Physical Solubility of CO_2_

The equilibrium between CO_2_ molecules present in gaseous and aqueous amine (i.e., PZ) solution measures the physical solubility of CO_2_.
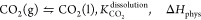
12It can be expressed by Henry’s law as follows
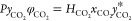
13where *P* represents system pressure, and *y*_CO_2__ and φ_CO_2__ represent the mole fraction and fugacity coefficient of CO_2_ in the vapor phase. *x*_CO_2__ and *y*_CO_2__^*^ are the equilibrium CO_2_ mole fraction and the asymmetric activity coefficient of CO_2_ in the solvent phase, respectively. The Poynting factor was taken as one. Henry’s Law constant of CO_2_ in water is represented by *H*_CO_2__ and has been extensively studied in the literature.^49,50^ A rigorous model based on all the literature data before 1991 (with pressures up to 1 MPa and temperature range 273 to 433 K) for a Henry’s constant of CO_2_ in water has been given by Carroll et al.^50^ Monteiro and Svendsen^51^ studied Henry’s constants of CO_2_ and N_2_O in water. They have emphasized that accurate Henry’s law constant correlations of CO_2_ and N_2_O in water are required to use the N_2_O analogy equation. They have also provided the latest modeling results with 95% confidence bands. In the present work, Henry’s law constant for CO_2_ in water is taken from the work of Carroll et al.^50^

The overall enthalpy of reaction of CO_2_ absorption, i.e., enthalpy associated with physical absorption of CO_2_ and enthalpy of various chemical reactions involved in the PZ–H_2_O–CO_2_ system, can then be expressed as
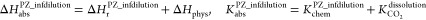
14corresponding to the reaction
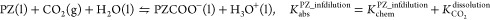
15

### 3.4 Heat of Absorption

Temperature-dependent enthalpies, entropies, and heat capacities can be calculated precisely using equilibrium reaction constants at different temperatures.^52−56^ In this work, temperature-dependent ln *K* values for different reactions based on eq 11 have been used to calculate standard enthalpy changes (Δ*H*) using the well-known Van’t Hoff’s equation. This equation can be easily derived from the Gibbs–Helmholtz equation.^57^
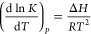
16Temperature-dependent ln *K* values can be expressed in the form given by Weiland et al.,^58^ as shown by the following equation
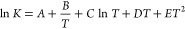
17Temperature-dependent Δ*H* is represented by eq 18, resulting by differentiating eq 17 w.r.t. temperature (*T*) following eq 16.
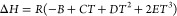
18

### 3.5 Parameter Fitting

Reaction equilibrium constants calculated using eq 11, as explained above in section 3.2, were fitted to eq 17 to obtain coefficients for the correlations, as given in Table 1. Temperature-dependent Δ*H* values for the different reactions were calculated by using eq 18, as explained in section 3.4.

**Table 1:** Coefficients for the Reaction Equilibrium Constants for Various Reactions in the PZ/CO_2_/H_2_O System Studied in This Work^a^

reaction no.	parameter	*A*	*B*	*C*	*D*	*E*	ref
(5)	*K*_a_^PZH_2_^2+^^	48525.05	–983443	–9213.91	28.80816	–0.01498	this work
(6)	*K*_a_^PZH^+^^	37.31499	–6091.49	–8.39402	0.033076	–1.69 × 10^–5^	this work
(8)	*K*_a_^H^+^PZCOO^–^^	454.1385	–9510.04	–88.4742	0.30593	–1.66 × 10^–4^	this work
(9)	*K*_c_^PZ(COO)_2_^2–^^	1501.275	–30477.6	–289.397	0.969801	–4.84 × 10^–4^	this work
(10)	*K*_c_^PZCOO^*–*^^ = *K*_chem_^PZ_infdilution^	–760.608	19823.69	143.7794	–0.51585	2.95 × 10^–4^	this work
(3)	*K*_HCO_3_^–^_	2005.822	–50154.6	–368.665	0.953995	–0.000 45	Kamps et al.^59^
(12)	*K*_CO_2__^dissolution^	–468.805	5284.795	95.04081	–0.32395	0.000 152	Edwards et al.^60^

a ln *K* = *A* + *B*/*T* + *C* ln *T* + *DT* + *ET*^2^, Δ*H* = *R*(−*B* + *CT* + *DT*^2^ + 2*ET*^3^). All equilibrium constants in the table are on mole fraction basis.

### 3.6 Free Energy of Solvation of Ionic Species and Temperature Dependence of Free Energy of Solution

The chemistry of the PZ/CO_2_/H_2_O system (reactions 5–9 given in section 3.2) contains many ionic species, and the free energy of solvation of ions calculated by employing the explicit solvation shell approach is considered to be more reliable as compared to continuum solvation models.^13,27,31,32,36,40−46^ Therefore, we have used the explicit solvation model introduced by da Silva et al.^35^ for calculating the free energy of solvation of ionic species in this work.

**Figure 1:**
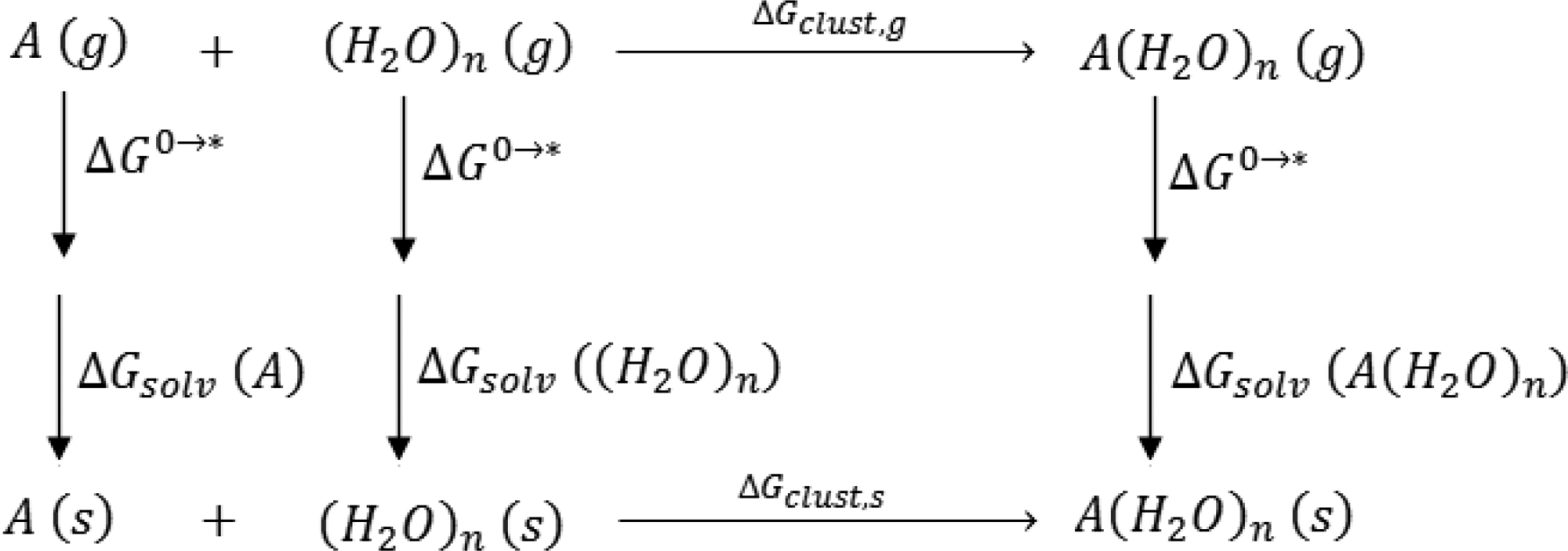
Thermodynamic cycle for calculating hydration free energies employing the explicit solvation shell model.

The literature has regularly reported the solvation free energies of ions employing cluster/continuum calculations using different thermodynamic cycles.^61−70^ The thermodynamic cycle for calculating solvation free energies using cluster-continuum models was previously explained in detail,^35,71^ and only a concise summary is provided here. Figure 1 presents the thermodynamic cycle used in this work.

Gaseous phase reactions between the solute (A) and water clusters (i.e., (H_2_O)_*n*_) are presented by the upper leg of the thermodynamic cycle (Figure 1).
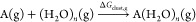
19The solvation free energy of the solute (Δ*G*_solv_^*^(A)) is computed from the thermodynamic cycle given in Figure 1 as follows:
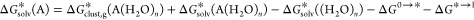
20The solvation free energy of the solute(Δ*G*_solv_^*^(A)) is given by the summation of the free energy of the gas phase solute–water cluster (Δ*G*_clust,g_^*^(A(H_2_O)_*n*_)), the difference between the solvation free energies of the solute–water cluster (Δ*G*_clust,g_^*^(A(H_2_O)_*n*_)) and the water cluster, Δ*G*_solv_^*^((H_2_O)_*n*_). The standard state corrections adjust the gas-phase concentrations (Δ*G*^0→*^ = *RT* ln(24.46)) from 1 mol per 24.46 L to 1 M and the water cluster concentration from 1 M to 55.34/nM^72,73^ (ΔG^*→l^ = *RT* ln([H_2_O])/*n*)). At room temperature, the gas-phase standard state correction (Δ*G*^0→*^) is 1.89 kcal mol^–1^.

In our previous work, we presented a method to evaluate the temperature dependency of the protonation reaction p*K*_a_ of various molecules (e.g., amines, alkanolamines, amino acids, carboxylic acids) within experimental error bars using computational chemistry tools.,^32,34,38^ In this method, the free energy of protonation in solution at 298 K is shifted to the experimental free energy of protonation in solution at 298 K (correction factor) following eq 21.
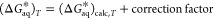
21

In this work, the solvation free energies at different temperatures are calculated using the SM8T solvation model, which provided amine basicity results consistent with experimental results, as discussed in section 2 above. We had also observed that, besides amine basicity, the results for the temperature dependency of the amine carbamate formation reaction were within experimental error bars computed for a data set of various PCC solvents.^3,33^ Therefore, the same approach is applied for calculating the temperature dependency of the deprotonation, carbamate formation, dicarbamate formation, and protonated carbamate formation reactions of piperazine with CO_2_. At 298 K, computed results are shifted to experimental values given by Ermatchkov et al.,^74^ and the temperature dependencies from computational chemistry results are retained. Gas-phase and solution-phase free energies of various species involved in the PZ/CO_2_/H_2_O system and corresponding correction factors applied are given in the Supporting Information (Table S1–S4).

## 4 Results and Discussion

Table 2 shows the calculated solvation free energies of different piperazine species in the PZ/CO_2_/H_2_O system calculated using explicit solvation shell model presented by da Silva et al.^35^ The cluster formation energies, entropies, and cluster solvation energies for protonated piperazine, diprotonated piperazine, piperazine carbamate, protonated piperazine carbamate, protonated piperazine carbamate, and piperazine dicarbamate studied in this work are also given in Table 2. The Poisson–Boltzmann continuum solvation model is used to calculate ESS cluster solvation energies presented in Table 2. All calculations and corrections to the final results were explained in the model proposed by da Silva et al.^35^ and are briefly presented in the thermodynamic framework section in this work.

**Table 2:** Free Energy of Solvation of Piperazine Species Involved in the PZ/CO_2_/H_2_O System Calculated by the Explicit Solvation Shell Model^a^

piperazine species	Δ*G*_solv_ (calcd)^b^	Δ*E*_cluster_^*^^c^	–*T*Δ*S*_cluster_^*^^d^	Δ*G*_s_ (*A*(*S*)_*n*_)^e^	area^f^
protonated piperazine (PZH^+^)	–59.1	–25.4	7.7	–53.82	242.62
diprotonated piperazine (PZH_2_^2+^)	–220.7	–68.5	10.1	–174.71	239.53
piperazine carbamate (PZCOO^–^)	–78.1	–38.7	10.8	–62.54	256.50
protonated piperazine carbamate (H^+^ PZCOO^–^)	–54.7	–34.8	13.0	–45.31	243.67
piperazine dicarbamate PZ(COO)_2_^2–^	–203.5	–58.1	11.2	–169.00	288.53

a All values are in kcal/mol.

b Calculated free energy of solvation; all values are shifted by −2.41 kcal/mol to remove systematic error relative to experimental values as in the ESS model presented by da Silva et al.^35^ The estimated sampling standard deviation is 1 kcal/mol.

c The energy of formation of the cluster at the HF/6-31+G (d) level, converted from a standard state of 1 atm to 1 mol/L. Thermal corrections to the energy and zero-point energies are not included.

d Temperature (298 K) multiplied by the entropy of formation of the cluster at the HF/6-31+G (d) level.

e Free energy of solvation of the cluster calculated with the Poisson–Boltzmann continuum model.

f Area of clusters calculated with the Poisson–Boltzmann continuum model.

**Figure 2:**
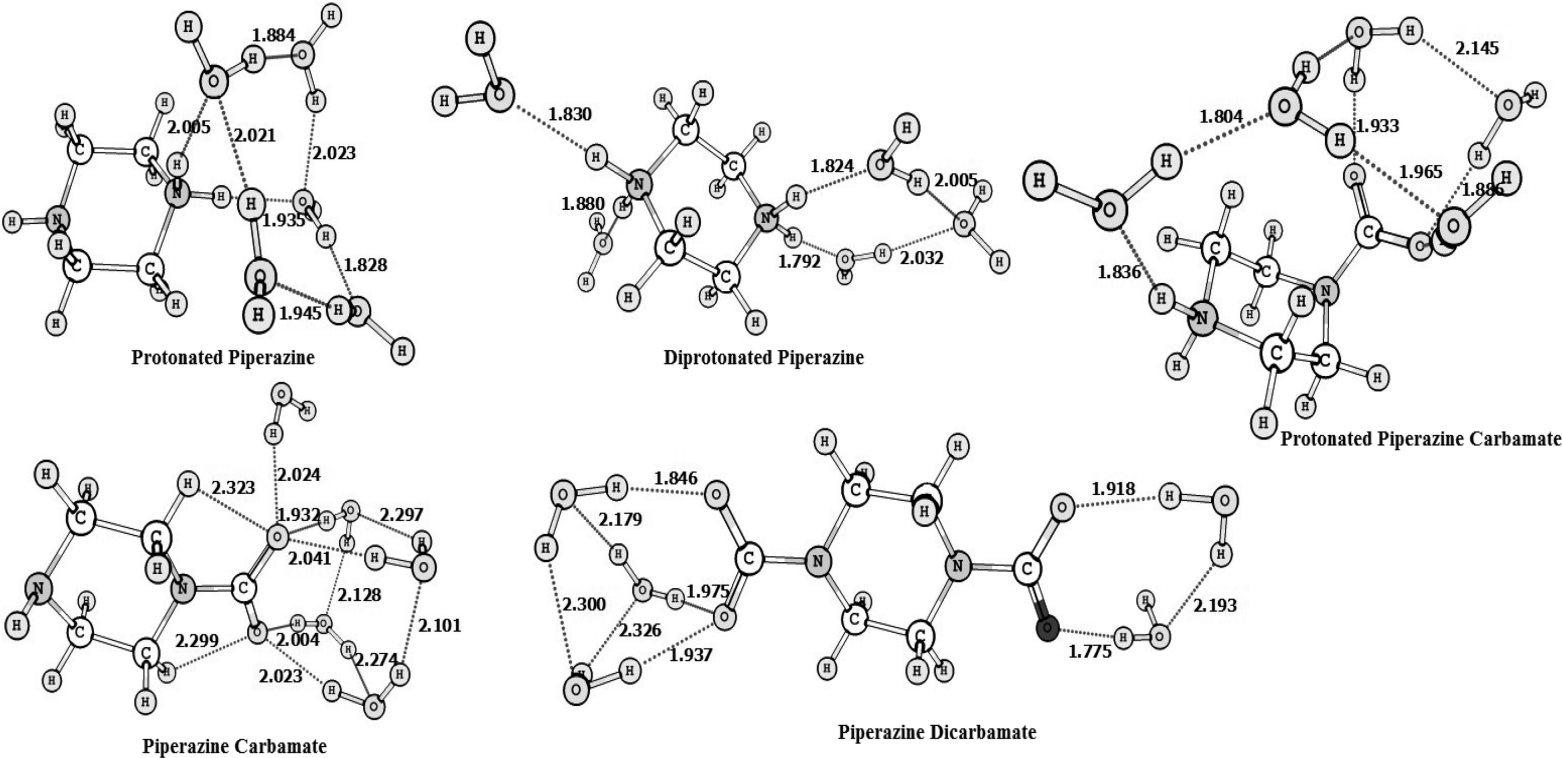
Optimized most stable ESS clusters of PZ species obtained in this work. (Dotted lines show hydrogen bonds, and hydrogen bond lenghths are given in angstroms).

The most stable optimized ESS clusters of protonated piperazine, deprotonated piperazine, protonated piperazine carbamate, piperazine carbamate, and piperazine dicarbamate with five explicit water molecules solvation shell are shown in Figure 2. The hydrogen bonds present within ESS clusters are also shown in this figure.

Table 3 lists gas-phase free energy and enthalpy of the different reactions occurring in the PZ/CO_2_/H_2_O system studied in this work at 298 K. From Table 3, we can see that results from using Gaussian–n theories (G3MP2B3, G3MP2, G4MP2, CBS-QB3) and density functional theories (DFT) at B3LYP/6-311++G (d,p) level are in the same range. Since gas-phase calculations using different methods agree well, DFT gas-phase results are used for the final calculations of reaction equilibrium constants of various reactions in the PZ/CO_2_/H_2_O system.

**Table 3:** Gas-Phase Free Energy and Enthalpy of Different Reactions Occurring in the PZ/CO_2_/H_2_O System Studied in This Work at 298 K^a^

reaction no.	G3MP2B3	G3MP2	G4MP2	CBS-QB3	DFT(B3LYP/6-311++G(d,p))
Gaseous Phase Free Energy of Reaction at 298 K					
(5)	–42.83	–42.85	–43.42	–43.34	–42.99
(6)	63.96	63.93	63.39	63.48	63.17
(8)	137.13	136.91	141.01	136.58	136.55
(9)	241.74	240.76	241.34	239.13	242.05
(10)	187.92	187.15	187.58	185.34	188.93
Gaseous Phase Enthalpy of Reaction at 298 K					
(5)	–42.75	–42.75	–43.32	–43.24	–42.81
(6)	63.68	63.61	63.13	63.23	62.43
(8)	136.51	136.32	141.16	136.04	136.10
(9)	230.07	229.58	229.67	227.37	230.34
(10)	176.41	176.07	176.12	173.75	177.68

a All values are in kJ/mol.

### 4.1 Deprotonation Reactions of PZH_2_^2+^, PZH^+^, and H^+^PZCOO^–^ (Reactions 5, 6, and 8, Respectively)

**Figure 3:**
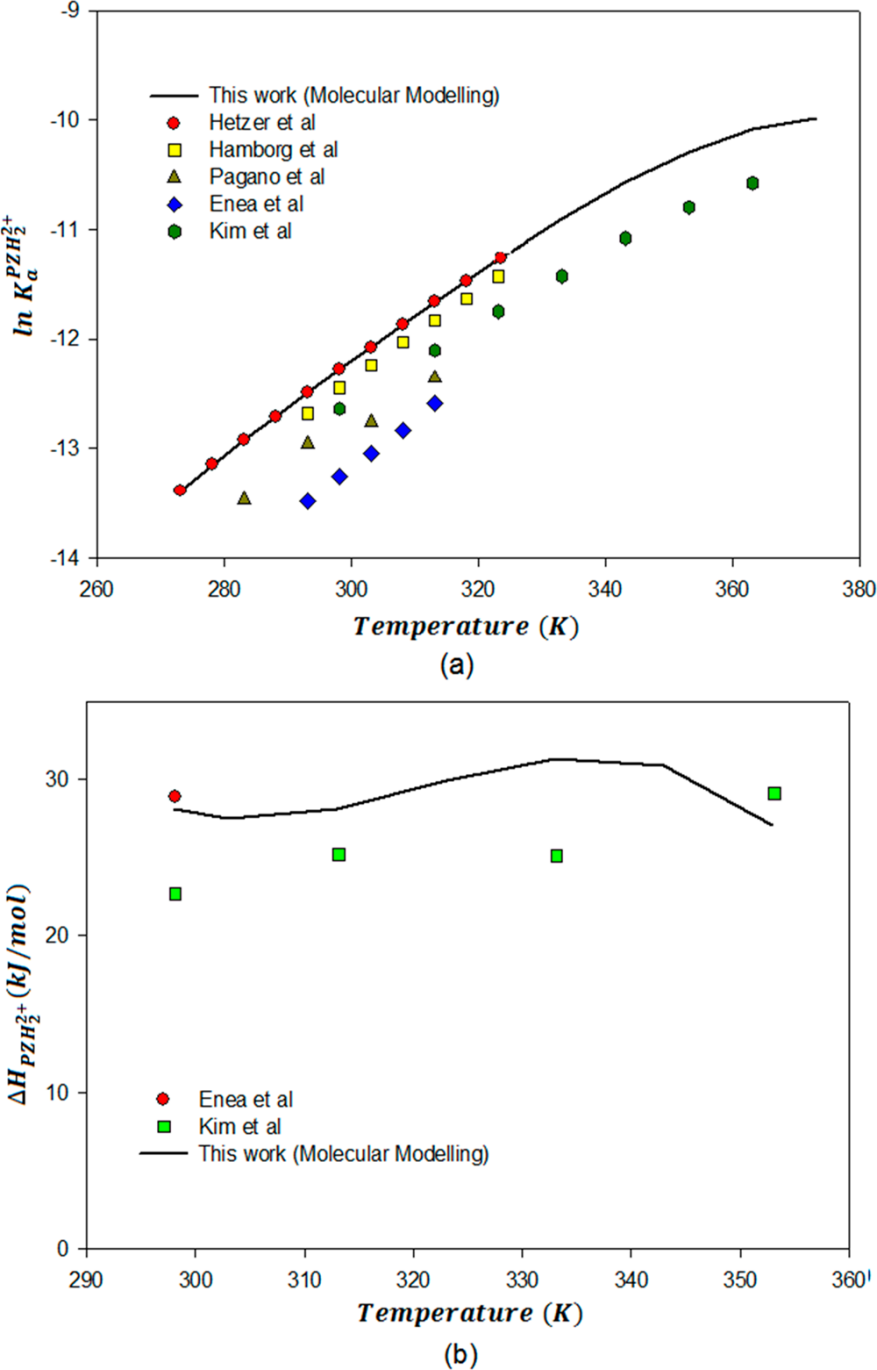
Dissociation constants (a) and enthalpies of deprotonation (b) for PZH_2_^2+^ as a function of temperature from molecular modeling compared with available literature data.

**Figure 4:**
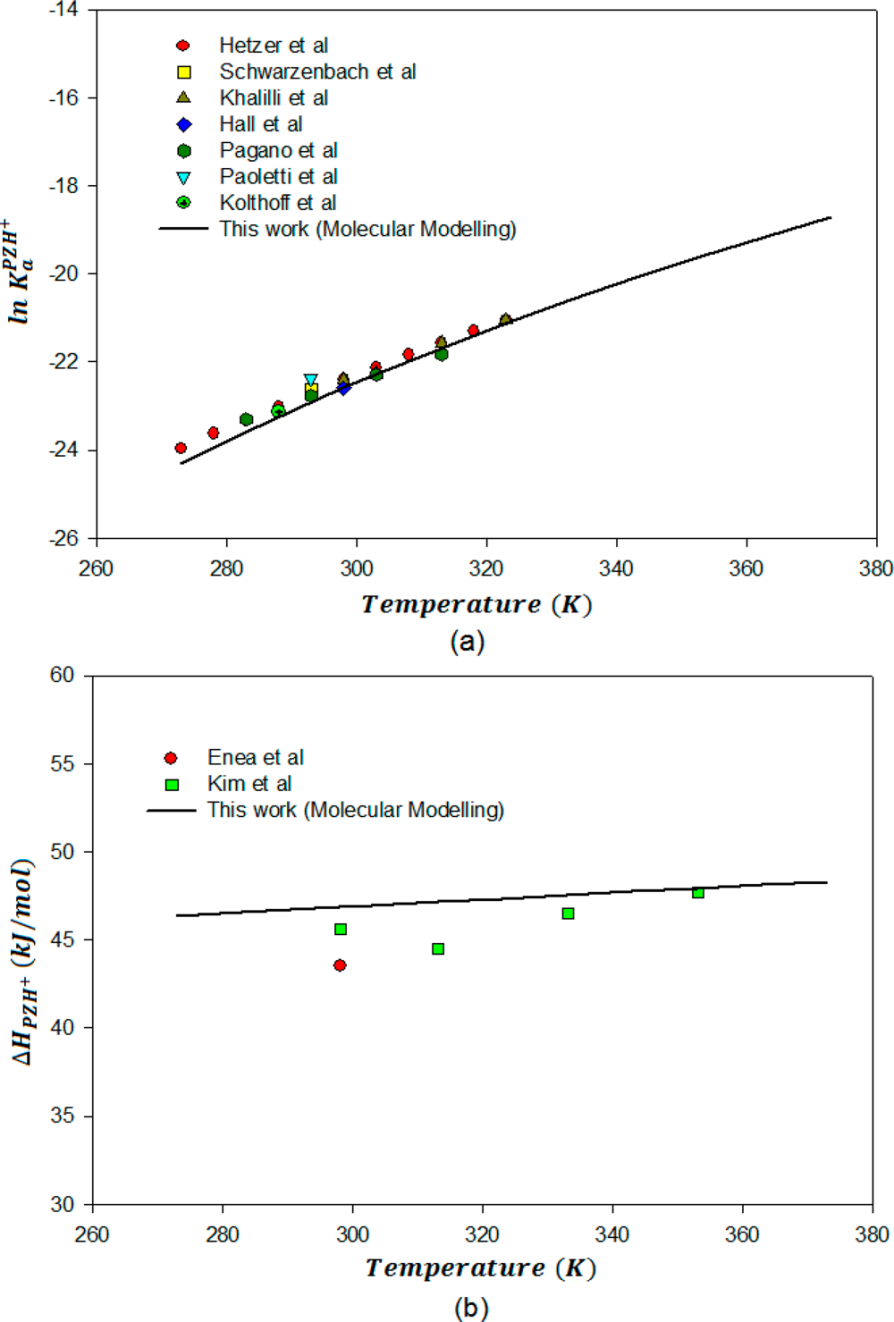
Dissociation constants (a) and enthalpies of deprotonation (b) for PZH^+^ as a function of temperature from molecular modeling compared with available literature data.

**Figure 5:**
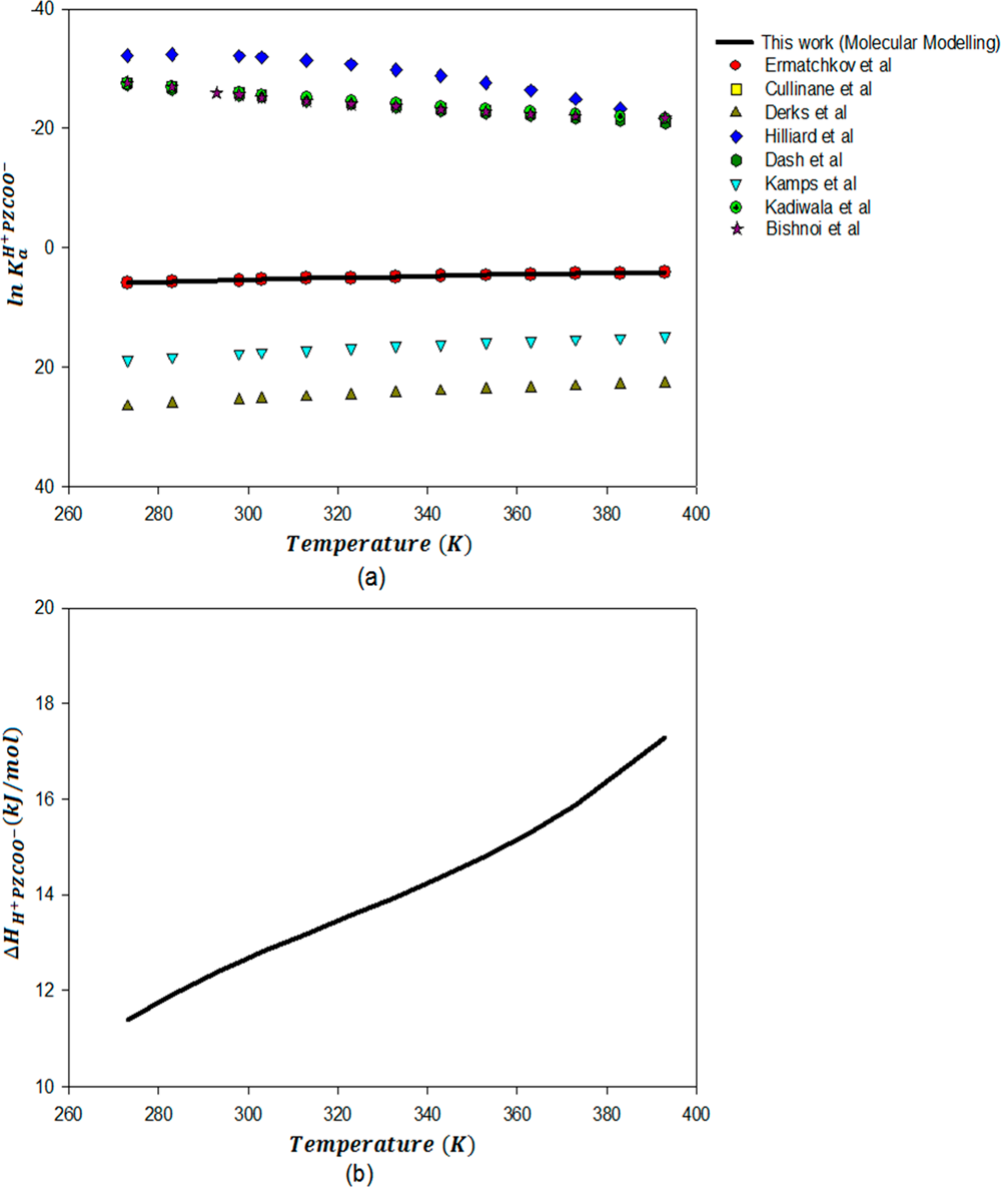
Dissociation constants (ln *K*_a_^H^+^PZCOO^–^^) (a) and enthalpies of deprotonation (Δ*H*_H^+^PZCOO^–^_) (b) for H^+^PZCOO^–^ as a function of temperature from molecular modeling compared with available literature data.

Dissociation constants for diprotonated piperazine, protonated piperazine, and protonated piperazine carbamate were calculated using the temperature-dependent SM8T and absolute ESS solvation free energy coupled with DFT quantum mechanical calculations in the gas phase. The temperature dependencies of dissociation constants of amines and alkanolamines are calculated within experimental error bars employing this method when taking experimental values of the free energy of protonation in solution at 298 K as input.^32^ Calculated temperature-dependent dissociation constants for PZH_2_^2+^, PZH^+^, and H^+^PZCOO^–^ along with available experimental results are plotted in Figures 3a, 4a, and 5a, respectively. Figure 3 shows that the temperature-dependent ln *K* values for the diprotonated and protonated piperazine deprotonation reactions are predicted within experimental error bars. There is a lot of variation in the experimental results for the dissociation constant corresponding to protonated piperazine carbamate at 298 K. However, the computed temperature dependency of the protonated piperazine carbamate dissociation constant agrees reasonably well with the experimental results given by Ermatchkov et al.^74^

As discussed in the methods section, enthalpies of protonation at different temperatures can be calculated using dissociation constants from the differentiation of the Van’t Hoff eq (eq 16). Figures 3b, 4b, and 5b present the calculated temperature-dependent enthalpies for deprotonation reactions of PZH_2_^2+^, PZH^+^, and H^+^PZCOO^–^, respectively. Available experimental data for enthalpy of these deprotonation reactions at different temperatures are also plotted.

The temperature-dependent enthalpies of deprotonation for PZH_2_^2^+^^ and PZH^+^ as given in Figures 3b and 4b, calculated using computational chemistry in present work, are well within experimental uncertainties. Experimentally measured data for the temperature-dependent deprotonation enthalpy of PZH_2_^2^+^^ and PZH^+^ are only given by Kim et al.^21^ (2011) and Enea et al.^75^ (1972). They had employed calorimetric experiments to measure the heat of absorption. Kim et al.^21^ have discussed that their determined first and second deprotonation reaction enthalpy values differ by 4.5% and 27.5%, respectively, from those of Enea et al.^75^ Although this discrepancy in the experimental results is slightly outside the range of experimental uncertainty, Kim et al.^21^ have discussed that this observed difference might be attributed to reaction conditions, experimental procedure, and calculation method. However, Figures 3b and 4b show that calculated heats of absorption for the first and second protonation constants of PZ in this work are predicted within the range of experimentally determined calorimetric heats of protonation. Figure 5b shows the temperature dependency of enthalpy of deprotonation of H^+^PZCOO^–^. However, there are no experimental data for the enthalpy of deprotonation of H^+^PZCOO^–^ as protonated piperazine carbamate is very difficult to individually speciate for measurement in the experimental system.

### 4.2 Carbamate and Dicarbamate Formation Reaction of PZ (Reactions 7 and 9)

**Figure 6:**
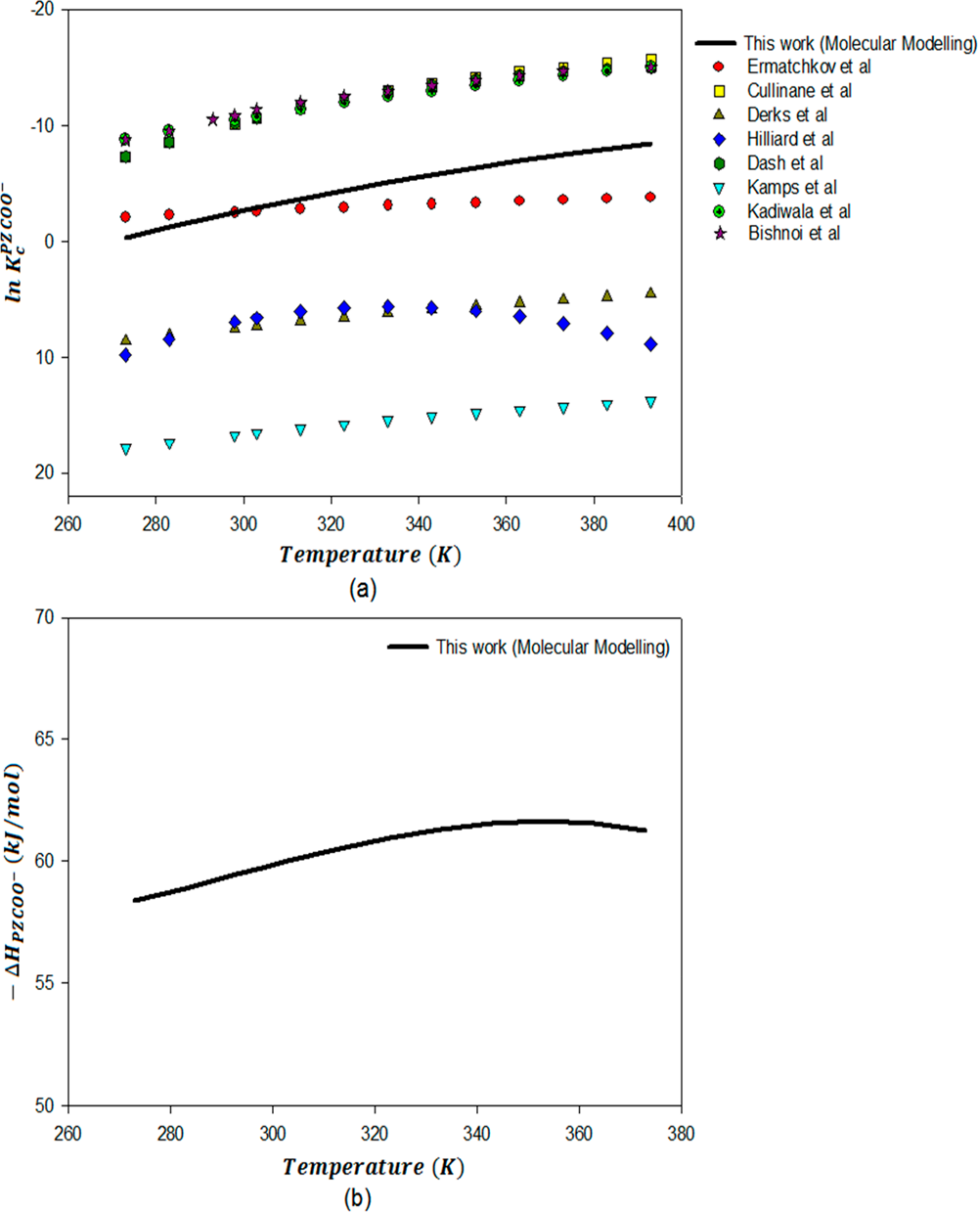
ln *K*_c_^PZCOO^–^^ (a) and −Δ*H*_PZCOO^–^_ (b) for PZ carbamate formation as a function of temperature (PZ(l) + CO_2_(l) + H_2_O(l) ⇋ PZCOO^–^(l) + H_3_O^+^(l)).

**Figure 7:**
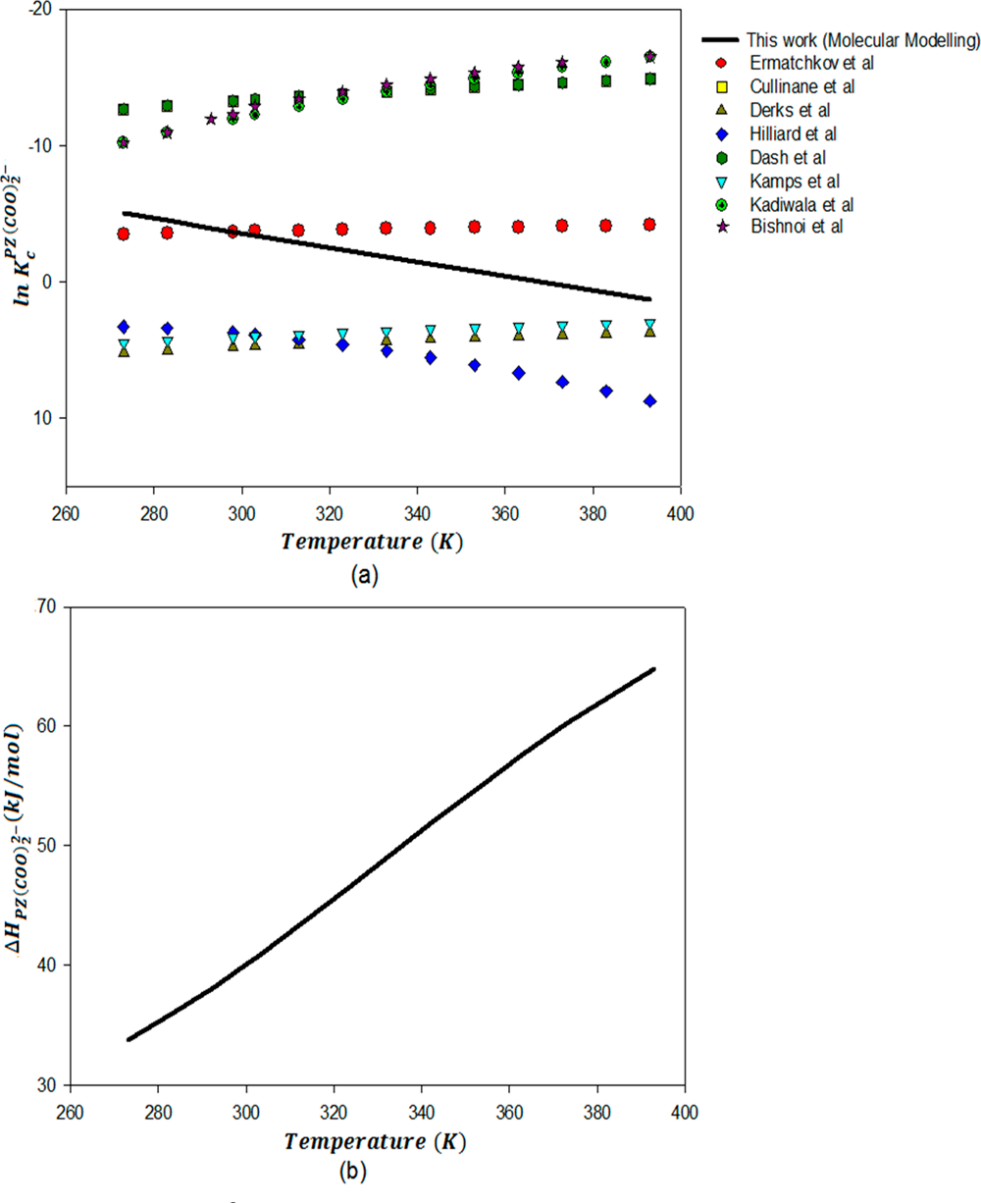
ln *K*_c_^PZ(COO)_2_^2–^^ (a) and Δ*H*^PZ(COO)_2_^2–^^ (b) for the PZ dicarbamate formation reaction as a function of temperature (PZCOO^–^(l) + CO_2_(l) + H_2_O(l) ⇋ PZ(COO)_2_^2–^)(l) + H_3_O^+^(l)).

Figures 6a and 7a present temperature-dependent equilibrium constant for piperazine carbamate formation (reaction 7) and piperazine dicarbamate reaction (reaction 9). From these figures, It can be seen that the data for ln *K*_c_^PZCOO–^ and ln *K*_c_^PZ(COO)_2_^2–^^ at 298 K is scattered based on various literature sources. The temperature dependency of these constants is also uncertain, as shown in graphs 6a and 7a, respectively. The only experimental data available for these constants for PZ are from the NMR work of Bishnoi et al.^9,10,76^ and Ermatchkov et al.^74^ Bishnoi^76^ gives estimates based on limited *P*_CO_2__^*^ and speciation data. Ermatchkov et al.^74^ presents constants regressed from a large amount of ^1^H NMR data using the Pitzer–Debye–Hückel model. There is also some literature that calculates concentration-based equilibrium constants from VLE data. Aroua and Salleh et al.^77^ regress the concentration-based equilibrium constants from VLE data. One of the complications about the experimental determination of these equilibrium constants is the ambiguity in the equilibrium constants of the different reactions because of constraints of current measurement devices. It is difficult to separate piperazine carbamate from protonated piperazine carbamate with NMR due to fast-changing proton transfer. So, measurements of their exact concentrations and subsequent calculation of their equilibrium constants is difficult. The calculation of temperature effects on *K*_carb_ of solvents of postcombustion CO_2_ capture employing computational chemistry can be considered as a valuable tool where experimental measurement of these constants through NMR spectroscopy or VLE is relatively uncertain and challenging. The computational chemistry values for ln *K*_c_ in this work are anchored to the experimental values measured by Ermatchkov et al.^74^ at 298 K. The equilibrium constants for the PZ carbamate and PZ dicarbamate reactions (reactions 7 and 9) calculated in this work are plotted as a function of temperature in Figures 6a and 7a along with available literature data.

There is no experimental data available in the literature for the enthalpy of the PZ carbamate and PZ dicarbamate formation reaction. Temperature-dependent enthalpies for the piperazine carbamate and piperazine dicarbamate formation reactions are calculated by application of Van’t Hoff’s eq (eq 16) to ln *K* correlations calculated from computational chemistry in this work. The piperazine carbamate and piperazine dicarbamate formation reaction enthalpies are plotted in Figures 6b and 7b, respectively.

### 4.3 Overall Differential Heat of Absorption of CO_2_ with Piperazine (Infinite Dilution) as a Function of Temperature at an Infinitely Low Loading of CO_2_ and PZ

In literature, the reaction mechanism of the PZ/CO_2_/H_2_O system is considered to consist of up to eight elementary reactions where reactions 2–9 were suggested by Bishnoi et al.,^10^ Ermatchkov et al.,^74^ and Hartono et al.^78^ In the present work, heats of absorption (Δ*H*_abs_), of the PZ/CO_2_/H_2_O system are calculated. The heat of absorption is given by the sum of the heat of dissolution and the heats of reaction as defined in section 3.1. The amount of CO_2_ absorbed in the amine solution is considered to react with the PZ in calculating the heat of absorption. Henry’s law is used to calculate the amount of physically dissolved carbon dioxide that has not chemically reacted with the amine solution. Therefore, the equilibrium constants (ln *K*) and enthalpy values for all reactions involved in the PZ/CO_2_/H_2_O system must be known to calculate overall heats of absorption. As discussed above, the temperature-dependent enthalpies of reactions 5–9 were determined from the SM8T and ESS solvation free energy values coupled with gas-phase DFT calculations. The reaction corresponding to the dissociation of carbon dioxide (i.e., reaction 3) is extensively studied in the literature. The temperature-dependent dissociation constant (ln *K*) for reaction three was taken from the work of Kamps et al.^59^ and the differential form of the Van’t Hoff equation was used to calculate the corresponding enthalpy. To calculate the overall enthalpy for the PZ/CO_2_/H_2_O system, the physical solubility of CO_2_ should also be added, as given by eq 12. Henry’s law constant for CO_2_ dissolution given by Edwards et al.^60^ was used in this work. Tables 4 and 5 provide the temperature-dependent ln *K* and enthalpy values of the various reactions involved in the PZ/CO_2_/H_2_O system calculated in this work in the temperature range 273.15–373 K.

**Table 4:** Temperature-Dependent ln *K* Values of Various Reactions Involved in the PZ/CO_2_/H_2_O System

temp (K)	ln *K*_a_^PZH_2_^2+^^	ln *K*_a_^PZH^+^^	ln *K*_a_^H^+^PZCOO^–^^	ln *K*_c_^PZ(COO)_2_^2–^^	ln *K*_c_^PZCOO^–^^ = ln *K*_chem_^PZ_infdilution^^a^	ln *K*_HCO_3_^–^_	ln *K*_CO_2__^dissolution^
273	–13.95	–24.31	–5.87	–5.03	–0.32	–19.14	6.61
283	–13.31	–23.59	–5.69	–4.49	–1.24	–18.89	6.95
298	–12.65	–22.59	–5.43	–3.68	–2.50	–18.63	7.39
303	–12.46	–22.27	–5.34	–3.42	–2.90	–18.58	7.51
313	–12.11	–21.68	–5.18	–2.88	–3.67	–18.51	7.74
323	–11.77	–21.12	–5.02	–2.35	–4.39	–18.47	7.94
333	–11.42	–20.59	–4.86	–1.81	–5.07	–18.47	8.10
343	–11.09	–20.08	–4.72	–1.28	–5.72	–18.51	8.24
353	–10.80	–19.61	–4.57	–0.75	–6.33	–18.56	8.36
363	–10.58	–19.16	–4.43	–0.22	–6.91	–18.64	8.46
373	–10.48	–18.73	–4.30	0.30	–7.47	–18.74	8.53
393	–10.81	–17.9344	–4.05	1.33	–8.45	–19.00	8.63

a Corresponds to reaction 10.

**Table 5:** Temperature-Dependent Enthalpy Values of Various Reactions Involved in the PZ/CO_2_/H_2_O System^a^

temp (K)	Δ*H*_1_	Δ*H*_2_	Δ*H*_4_	Δ*H*_5_	Δ*H*_HCO_3_^–^_	Δ*H*_phys_	Δ*H*_3_ = Δ*H*_r_^PZ_infdilution^^b^	Δ*H*_abs_^PZ_infdilution^^c^
273	47.42	46.38	11.53	33.78	18.48	–22.62	–58.37	–80.99
283	35.67	46.56	11.91	35.89	14.51	–21.43	–58.90	–80.33
298	28.10	46.85	12.53	39.51	9.12	–19.43	–59.72	–78.45
303	27.52	46.95	12.75	40.82	7.45	–18.73	–59.99	–77.24
313	28.15	47.15	13.19	43.53	4.27	–17.25	–60.51	–76.22
323	29.91	47.35	13.61	46.34	1.27	–15.71	–60.96	–75.09
333	31.32	47.55	14.02	49.20	–1.60	–14.13	–61.31	–73.82
343	30.87	47.75	14.37	52.07	–4.38	–12.51	–61.55	–72.43
353	27.09	47.94	14.67	54.90	–7.13	–10.88	–61.63	–70.87
363	18.46	48.14	14.89	57.64	–9.88	–9.24	–61.54	–69.16
373	3.51	48.32	15.01	60.24	–12.68	–7.62	–61.23	–65.71
393	–51.37	48.67	14.91	64.82	–18.61	–4.48	–59.87	–59.87

a Δ*H*_1_, Δ*H*_2_, and Δ*H*_4_ correspond to the enthalpy of deprotonation of PZH_2_^2^+^^, deprotonation of PZH^+^, and deprotonation of H^+^PZCOO^–^, respectively. Δ*H*_3_ = Δ*H*_r_^PZ_infdilution^ corresponds to the enthalpy of carbamate formation reaction of PZ, Δ*H*_5_ corresponds to the enthalpy of dicarbamate formation of the PZ reaction. The enthalpy of dissociation of carbon dioxide is represented by Δ*H*_HCO_3_^–^_, and Δ*H*_phys_ corresponds to the enthalpy of physical solubility of CO_2_. Deprotonation of PZH_2_^2^+^^, deprotonation of PZH^+^, deprotonation of H^+^PZCOO^–^, carbamate formation reaction of PZ, dicarbamate formation of PZ reaction, dissociation of carbon dioxide, and physical solubility of CO_2_ are represented by eqs 5, 6, 8, 7, 9, 3, and 12, respectively.

b Δ*H*_r_^PZ_infdilution^ is calculated by correlating ln *K*_Chem_^PZ_infdilution^ corresponding to ln *K*_c_^PZCOO^–^^ values from the Gibbs–Helmholtz equation.

c Δ*H*_abs_^PZ_infdilution^ = Δ*H*_r_^PZ_infdilution^ + Δ*H*_phys_.

The Piperazine carbamate formation reaction (reaction 7) and piperazine dicarbamate formation reaction (reaction 9) mainly govern the heat of reaction of piperazine absorption in the PZ/CO_2_/H_2_O system.^79^ As discussed by Liu et al.,^48^ at low CO_2_ loadings, the carbamate formation reaction of PZ (reaction 7) determines the heat of absorption of reaction, but, as the reaction proceeds, the concentration of free piperazine decreases and reaction 9, i.e., the formation of piperazine dicarbamate starts to dominate the heat of absorption. The total heat of absorption and heats of each individual reaction of the PZ/CO_2_/H_2_O system at 313 K using the eNRTL model has been given by Hartono et al.^78^ They have shown that, at low loadings of up to 0.6, that the heat of absorption is mainly governed by formation of piperazine carbamate, but, at CO_2_ loading higher than 0.6, protonated piperazine carbamate becomes the main species. In this work, we also have conditions of infinitely low loading of piperazine and CO_2_ and infinite dilution. Under such conditions, the carbamate formation reaction of PZ (reaction 7) will govern the heat of absorption of the system in an aqueous solution and is termed Δ*H*_3_ = Δ*H*_r_^PZ_infdilution^. Physical absorption of CO_2_ is added to Δ*H*_r_^PZ_infdilution^ to give the overall Δ*H*_abs_^PZ_infdilution^ (Δ*H*_abs_^PZ_infdilution^ = Δ*H*_r_^PZ_infdilution^ + Δ*H*_phys_) according to eq 14.

**Figure 8:**
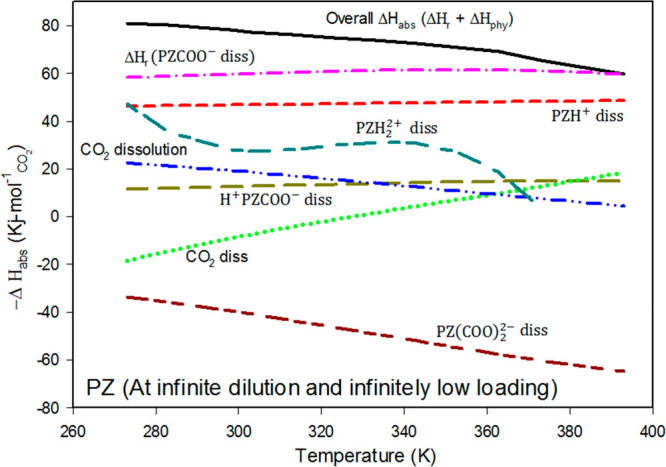
Overall differential heat of absorption of CO_2_ with PZ (infinite dilution solution) for the reaction PZ(l) + CO_2_(l) + H_2_O(l) ⇋ PZCOO^–^(l) + H_3_O^+^(l) and heats of each of the individual reactions as a function of temperature at infinite dilution and infinitely low loading of CO_2_.

Figure 8 presents the heats of reaction for each of the individual reactions between piperazine and CO_2_ at infinite dilution in the temperature range of 273.15 to 373. Δ*H*_r_ and Δ*H*_abs_ represent the heat of reaction in the solution phase and the overall heat of reaction of the PZ/CO_2_/H_2_O system, respectively.

The average Δ*H*_abs_ obtained in the temperature range 273.15–373 K in this work was 73.77 kJ/mol. In the recent study by Liu et al.,^79^ the heats of absorption for the low CO_2_ loading interval (<0.5 mol-CO_2_/mol-Am), the values are around 72–74 kJ/mol CO_2_ at all temperatures. The values of heat of absorption calculated by Cullinane et al.^18^ and Xu et al.^80^ using the Gibbs–Helmholtz equation based on the VLE data given for different temperatures and PZ concentrations from 0.9 to 12 mol, were around 66–69 kJ/mol CO_2_. However, the effect of temperature on the heat of absorption was disregarded in their calculations. Hilliard et al.^16^ and Kim et al.^24^ used a reaction calorimeter to directly measure the heat of absorption of CO_2_ into a 2.4 mol PZ solution. The values for the heat of absorption measured by them were around 70–75 kJ/mol. Calculations by Cullinane and Rochelle^18^ using the Gibbs–Helmholtz equation based on the VLE data for PZ show a marked decrease in the heat of absorption with increasing temperature. At the same time, a recent study from Svensson et al.^13^ found a slightly increasing heat of absorption of CO_2_ in PZ with an increase in temperature. However, both the data from the present study and those from the literature show that the enthalpy of absorption in PZ/CO_2_/H_2_O system is temperature dependent. However, the temperature dependency in the PZ/CO_2_/H_2_O system is weak as compared to that in the MEA/CO_2_/H_2_O system.

Compared with available literature data, we can see that the calculated Δ*H*_abs_ and its temperature dependency is within the experimental error bars. Computational chemistry provides a valuable tool for making predictions for Δ*H*_abs_ of various amine and alkanolamine solvents for PCC in water.

## 5 Conclusions

The computational methodology presented in this work can accurately estimate temperature-dependent enthalpy of the absorption of the PZ/CO_2_/H_2_O system and of involved individual deprotonation and carbamate formation reactions. The results of temperature-dependent enthalpy of absorption of CO_2_ in piperazine determined in the present work show that computational chemistry can be employed as an efficient tool to screen PCC solvents. These computational methods of determining the enthalpy of absorption of PCC solvents also become essential as experimental data of equilibrium constants and enthalpy of both overall and individual reactions involving highly reactive and short-lived species is difficult and relatively expensive. The computational chemistry methods used in this work for determining temperature-dependent enthalpy provide the opportunity to screen a large data set of potential PCC solvents efficiently. The average Δ*H*_abs_ obtained in the temperature range 273.15–373 K in this work is 73.77 kJ/mol. However, the predominant result of the present study is that the enthalpy of absorption of CO_2_ into PZ solutions is temperature-dependent, as calculated from the fundamental Gibbs–Helmholtz equation. With an increase in temperature, Δ*H*_abs_ of the PZ/CO_2_/H_2_O system become less exothermic, and therefore, it is easier to remove CO_2_ in the stripper column. Also, it should be noted that to calculate the temperature dependency of the enthalpy of the overall absorption reaction, specific temperature dependencies for the deprotonation and carbamate formation constants are required as input. Experimental determination of various equilibrium constants such as carbamate and deprotonation constants of amines and alkanolamines at high temperatures is challenging. Temperature-dependent Δ*H*_abs_ at infinite dilution calculated using computational chemistry can provide molecular-level insight into the chemistry of individual speciation and reactions involved to evaluate a PCC solvent’s overall performance. The results and correlations given in this work can be utilized in thermodynamic modeling (e.g., eNRTL, e-UNIQUAC)^81,82^ to predict the absorption of CO_2_ into other amines and alkanolamines essential for CO_2_ capture processes for the future development of PCC solvents.

## Supporting Information Available

Gaseous phase Gibbs free energy and enthalpy of piperazine species, using G3MP2B3, G3MP2, G4MP2, CBS-QB3, and DFT level of theories, at 298 K. Gaseous phase Gibbs free energy and enthalpy of bicarbonate (HCO_3_^–^), water (H_2_O), CO_2_, and H_3_O^+^ using G3MP2B3, G3MP2, G4MP2, CBS-QB3, and DFT level of theories, at 298 K. Underlying data for Table 2 for piperazine species: energy of solute and cluster in gas phase, thermal corrections to the energy and entropy of solute and cluster. Quantitative details of optimized structures of the first solvation shell from molecular simulations for a set of 100 initial geometries of piperazine. Molecular geometries of ESS clusters for various piperazine species. Simulation details. Explanation of QM/PB continuum solvent model. Constant terms utilized in calculations for ESS model. Full citation for references used in the manuscript (PDF)
