# Supplementary material for: Modeling Differential Enthalpy of Absorption of CO2 with Piperazine as a Function of Temperature
Source: J Phys Chem B. 2022 Feb 28;126(9):1980–91. doi: 10.1021/acs.jpcb.1c10755 (PMC8919260; doi:10.1021/acs.jpcb.1c10755)
Supplement: Supplementary file 1 — jp1c10755_si_001.pdf [file jp1c10755_si_001.pdf]

Supporting Information

Modeling Differential Enthalpy of Absorption of CO_2 with Piperazine as a Function of Temperature

Mayuri Gupta[§,a], Eirik Falck da Silva[b], Hallvard F. Svendsen[,a]*

[a]Department of Chemical Engineering, Norwegian University of Science and Technology, Trondheim 7491, Norway.

[b]Department of Process Technology, SINTEF Industry, Trondheim 7034, Norway

Prepared for ***The Journal of Physical Chemistry B***

Number of Pages: 17

Correspondence:
Prof. Hallvard F. Svendsen
Email: hallvard.svendsen@ntnu.no

Table of Contents

Description	Pages
Gaseous Phase Gibbs free energy of piperazine species, using G3MP2B3, G3MP2, G4MP2, CBS-QB3 and DFT level of theories, at 298 K.	S3
Gaseous Phase enthalpy of piperazine species, using G3MP2B3, G3MP2, G4MP2, CBS-QB3 and DFT level of theories, at 298 K.	S3
Gaseous Phase Gibbs free energy and enthaply of bicarbonate (HCO_3^-), water (H_2O), CO_2 and H_3O^+ using G3MP2B3, G3MP2, G4MP2, CBS-QB3 and DFT level of theories, at 298 K.	S4
Underlying data for Table 2 for piperazine species: energy of solute and cluster in gas phase, thermal corrections to the energy and entropy of solute and cluster.	S4
Quantitative details of optimized structures of the first solvation shell from molecular simulations for a set of 100 initial geometries of Piperazine.	S5 – S7
Molecular Geometries of ESS clusters for various Piperazine species	S8 – S12
Simulation Details	S13
Explanation of QM/PB Continuum Solvent Model	S14 – S15
Constant terms utilized in calculations	S16
Full citation for References used in manuscript	S16 – S17

Table S1: Gaseous Phase Gibbs free energy of piperazine species, using G3MP2B3, G3MP2, G4MP2, CBS-QB3 and DFT level of theories, at 298 K. (All values are in Hartrees)

Amine	G3MP2B3	G3MP2	G4MP2	CBS-QB3	DFT(B3LYP/6-311++G(d,p))
Piperazine (PZ)	-267.5044	-267.4909	-267.5300	-267.4474	-267.8927
Protonated Piperazine (PZH^+)	-267.8644	-267.8512	-267.8900	-267.8064	-268.2527
Diprotonated Piperazine (PZH_2^{2+})	-268.0543	-268.0413	-268.0798	-267.9951	-267.9951
Piperazine Carbamate ($PZCOO^-$)	-455.3540	-455.3337	-455.4021	-455.2877	-455.9889
Protonated Piperazine Carbamate (H^+PZCOO^-)	-455.8307	-455.8103	-455.8858	-455.7632	-456.4658
Piperazine Dicarbamate $PZ(COO)_2^{2-}$	-643.1179	-643.0911	-643.1886	-643.0422	-644.0004

Table S2: Gaseous Phase enthalpy of piperazine species, using G3MP2B3, G3MP2, G4MP2, CBS-QB3, DFT and HF level of theories, at 298 K. (All values are in Hartrees)

Amine	G3MP2B3	G3MP2	G4MP2	CBS-QB3	DFT(B3LYP/6-311++G(d,p))
Piperazine (PZ)	-267.4692	-267.4557	-267.4949	-267.4123	-267.8580
Protonated Piperazine (PZH^+)	-267.8290	-267.8157	-267.8547	-267.7712	-268.2170
Diprotonated Piperazine (PZH_2^{2+})	-268.0192	-268.0063	-268.0449	-267.9603	-267.9951
Piperazine Carbamate ($PZCOO^-$)	-455.3119	-455.2917	-455.3601	-455.2459	-455.9470
Protonated Piperazine Carbamate (H^+PZCOO^-)	-455.7878	-455.7676	-455.8443	-455.7208	-456.4234
Piperazine Dicarbamate $PZ(COO)_2^{2-}$	-643.0691	-643.0424	-643.1401	-642.9940	-643.9521

Table S3: Gaseous Phase Gibbs free energy and enthaply of bicarbonate (HCO_3^-), water (H_2O), CO_2 and H_3O^+ using G3MP2B3, G3MP2, G4MP2, CBS-QB3, DFT and HF level of theories, at 298 K. (All values are in Hartrees)

Species	G3MP2B3	G3MP2	G4MP2	CBS-QB3	DFT(B3LYP/6-311++G(d,p))
Bicarbonate	-264.2052	-264.1949	-264.2390	-264.1876	-264.5509
Water	-76.3633	-76.3600	-76.3735	-76.3551	-76.4549
CO_2	-76.6214	-76.6184	-76.6325	-76.6129	-76.7142
H_3O^+	-188.4072	-188.3995	-188.4301	-188.3934	-188.6566
**Gaseous phase Enthalpy**					
Bicarbonate	-264.1749	-264.1647	-264.2087	-264.1573	-264.5210
Water	-76.3419	-76.3386	-76.3521	-76.3337	-76.4335
CO_2	-76.6002	-76.5973	-76.6113	-76.5918	-76.6930
H_3O^+	-188.3822	-188.3753	-188.4052	-188.3685	-188.6317

Table S4: Underlying data for Table 2 for Piperazine species: energy of solute and cluster in gas phase, thermal corrections to the energy and entropy of solute and cluster.

Amine	$E^a(A)$ [a.u.]	$E^a(A(S)_5)$ [a.u.]	$E_{corr}{}^b(A)$ [a.u.]	$E_{corr}{}^b(A(S)_5)$ [a.u.]	$S_{total}{}^c(A)$ [cal/mol]	$S_{total}{}^c(A(S)_5)$ [cal/mol]
Piperazine (PZ)	-266.17	-646.32	0.16	0.31	72.37	158.61
Protonated Piperazine (PZH^+)	-266.56	-646.73	0.18	0.33	72.87	161.63
Diprotonated Piperazine (PZH_2^{2+})	-266.77	-647.01	0.20	0.34	73.38	154.31
Piperazine Carbamate ($PZCOO^-$)	-453.25	-833.45	0.17	0.32	85.90	164.29
Protonated Piperazine Carbamate (H^+PZCOO^-)	-453.75	-833.93	0.18	0.33	87.12	158.29
Piperazine Dicarbamate PZ $(COO)_2^{2-}$	-640.25	-1020.48	0.17	0.32	98.89	175.98

a: Energy of solute and solute-solvent cluster, standard state of 1 atmosphere. b: Thermal correction to the energy and zero-point energy for solute and solute-solvent cluster, standard state of 1 atmosphere. c: Total entropy of solute and solute-solvent cluster, standard state of 1 atmosphere.

Table S5: Quantitative details of optimized structures of the first solvation shell from molecular simulations for a set of 100 initial geometries of Piperazine.

<table>
<tr><th>Energy</th><th>Total Entropy</th><th>Trans Ent</th><th>Rot Ent</th><th>Vib Ent</th><th>dGsolv</th><th>Area</th></tr>
<tr><td>-646.305</td><td>163.72</td><td>41.405</td><td>32.617</td><td>89.697</td><td>-22.37</td><td>245.31</td></tr>
<tr><td colspan="7">Ignoring Geometry</td></tr>
<tr><td>-646.319</td><td>159.891</td><td>41.405</td><td>32.678</td><td>85.807</td><td>-18.91</td><td>243.3268</td></tr>
<tr><td>-646.294</td><td>148.909</td><td>41.405</td><td>32.527</td><td>74.976</td><td>-22.72</td><td>237.7864</td></tr>
<tr><td colspan="7">Ignoring Geometry</td></tr>
<tr><td>-646.317</td><td>169.288</td><td>41.405</td><td>32.647</td><td>95.235</td><td>-18.83</td><td>241.1956</td></tr>
<tr><td>-646.295</td><td>157.396</td><td>41.405</td><td>32.604</td><td>83.387</td><td>-24.67</td><td>245.8227</td></tr>
<tr><td>-646.298</td><td>172.153</td><td>41.405</td><td>32.663</td><td>98.084</td><td>-23.99</td><td>247.86</td></tr>
<tr><td colspan="7">Ignoring Geometry</td></tr>
<tr><td>-646.322</td><td>158.088</td><td>41.405</td><td>32.198</td><td>84.484</td><td>-16.29</td><td>230.4662</td></tr>
<tr><td>-646.325</td><td>158.613</td><td>41.405</td><td>32.216</td><td>84.992</td><td>-15.62</td><td>228.4892</td></tr>
<tr><td>-646.319</td><td>165.669</td><td>41.405</td><td>32.651</td><td>91.612</td><td>-19.19</td><td>243.2776</td></tr>
<tr><td>-646.324</td><td>153.053</td><td>41.405</td><td>32.397</td><td>79.25</td><td>-16.4</td><td>236.1716</td></tr>
<tr><td>-646.325</td><td>155.324</td><td>41.405</td><td>32.357</td><td>81.562</td><td>-15.82</td><td>235.5874</td></tr>
<tr><td>-646.325</td><td>160.946</td><td>41.405</td><td>32.338</td><td>87.203</td><td>-15.8</td><td>237.753</td></tr>
<tr><td>-646.325</td><td>161.02</td><td>41.405</td><td>32.334</td><td>87.28</td><td>-15.65</td><td>235.8431</td></tr>
<tr><td>-646.306</td><td>157.32</td><td>41.405</td><td>32.435</td><td>83.48</td><td>-20.68</td><td>235.0066</td></tr>
<tr><td colspan="7">Ignoring Geometry</td></tr>
<tr><td colspan="7">Ignoring Geometry</td></tr>
<tr><td>-646.304</td><td>155.833</td><td>41.405</td><td>32.947</td><td>81.48</td><td>-22.83</td><td>250.3674</td></tr>
<tr><td>-646.325</td><td>157.494</td><td>41.405</td><td>32.572</td><td>83.517</td><td>-16.61</td><td>242.7598</td></tr>
<tr><td>-646.315</td><td>157.256</td><td>41.405</td><td>32.428</td><td>83.423</td><td>-17.76</td><td>229.2114</td></tr>
<tr><td colspan="7">Ignoring Geometry</td></tr>
<tr><td>-646.325</td><td>158.564</td><td>41.405</td><td>32.489</td><td>84.67</td><td>-16.37</td><td>239.0556</td></tr>
<tr><td>-646.319</td><td>156.669</td><td>41.405</td><td>32.442</td><td>82.822</td><td>-15.5</td><td>237.5191</td></tr>
<tr><td>-646.306</td><td>167.729</td><td>41.405</td><td>33.111</td><td>93.212</td><td>-23.13</td><td>258.3611</td></tr>
</table>

-646.325	160.62	41.405	32.357	86.858	-15.8	236.8855
-646.316	170.457	41.405	32.72	96.331	-18.58	243.5136
-646.316	169.643	41.405	32.788	95.45	-18.76	244.1683
-646.314	166.851	41.405	32.739	92.706	-19.68	240.333
-646.323	163.496	41.405	32.248	89.843	-15.77	229.0635
-646.321	158.884	41.405	32.572	84.906	-16.67	237.8891
-646.314	171.654	41.405	32.556	97.693	-17.73	237.6735
-646.324	159.213	41.405	32.356	85.451	-16.28	235.7552
-646.323	159.862	41.405	32.441	86.015	-16.57	231.4826
-646.325	161.878	41.405	32.361	88.112	-15.89	234.388
-646.305	167.522	41.405	32.532	93.585	-21.88	244.2001
Ignoring Geometry						
-646.303	170.672	41.405	32.952	96.315	-23.19	255.3403
Ignoring Geometry						
Ignoring Geometry						
Ignoring Geometry						
Ignoring Geometry						
-646.311	161.21	41.405	32.45	87.354	-18.71	240.2328
-646.318	156.708	41.405	32.641	82.662	-18.02	244.358
-646.315	141.846	41.405	32.499	67.942	-17.49	238.1246
-646.307	146.326	41.405	32.52	72.401	-21.06	239.5587
-646.32	150.774	41.405	32.118	77.251	-15.32	225.0475
-646.324	155.405	41.405	32.261	81.739	-16.31	229.2547
-646.319	166.572	41.405	32.653	92.513	-19.1	242.733
-646.321	160.461	41.405	32.273	86.783	-17.65	231.7141
-646.305	145.376	41.405	32.519	71.452	-21.12	239.6916
-646.323	155.253	41.405	32.238	81.61	-16.11	229.8233
-646.318	160.085	41.405	33.036	85.644	-19.07	245.2934
Ignoring Geometry						
Ignoring Geometry						
-646.323	147.587	41.405	32.438	73.743	-17.26	239.9283
-646.309	147.564	41.405	32.389	73.769	-20.33	235.3033
-646.326	157.726	41.405	32.538	83.782	-16.39	241.7118
-646.32	156.62	41.405	32.428	82.786	-15.96	235.0708
-646.325	150.803	41.405	32.539	76.858	-16.75	240.1498
-646.316	132.908	41.405	32.678	58.824	-18.91	244.6796
-646.325	161.305	41.405	32.334	87.566	-15.74	237.469
-646.321	159.668	41.405	32.609	85.654	-17.17	239.4851

-646.318	149.127	41.405	32.364	75.357	-17.9	233.5917
-646.314	159.774	41.405	32.449	85.92	-19.38	238.6099
-646.317	162.344	41.405	32.257	88.682	-18.06	229.9863
-646.324	163.612	41.405	32.333	89.874	-16.05	235.9243
-646.312	145.752	41.405	32.442	71.905	-19.88	238.7577
-646.325	153.77	41.405	32.269	80.095	-15.41	228.3829
Ignoring Geometry						
-646.316	161.571	41.405	32.725	87.441	-18.59	243.4493
-646.319	140.073	41.405	32.331	66.336	-16.94	236.405
-646.317	168.667	41.405	32.733	94.529	-18.09	245.6794
Ignoring Geometry						
-646.316	163.947	41.405	32.718	89.823	-18.55	243.5719
-646.318	168.933	41.405	32.544	94.983	-17.82	238.2395
Ignoring Geometry						
-646.323	161.505	41.405	32.337	87.763	-15.99	231.2174
-646.308	152.546	41.405	32.145	78.996	-17.57	224.8564
-646.315	166.811	41.405	33.002	92.404	-18.46	245.7989
Ignoring Geometry						
-646.323	160.65	41.405	32.306	86.938	-15.69	234.092
-646.314	157.047	41.405	32.645	82.997	-18.14	243.8358
-646.312	163.127	41.405	32.915	88.807	-19.72	246.7401
-646.318	148.439	41.405	32.854	74.179	-17.92	241.6334
-646.317	148.034	41.405	32.605	74.024	-17.34	243.2641
-646.318	161.229	41.405	32.63	87.193	-19.19	239.1302
-646.319	169.929	41.405	32.554	95.969	-18.53	243.5605
-646.317	158.319	41.405	32.248	84.665	-19.46	228.6538
-646.308	164.258	41.405	32.287	90.566	-20.3	230.6688
-646.319	163.935	41.405	32.653	89.877	-19.06	242.4504
-646.321	156.593	41.405	32.571	82.616	-16.66	239.2248
-646.3	154.608	41.405	32.858	80.345	-22.89	251.3768
Ignoring Geometry						
-646.294	149.73	41.405	32.454	75.871	-23.48	241.896
-646.297	158.089	41.405	32.8	83.884	-23.92	250.8131
-646.317	166.152	41.405	32.502	92.245	-18.61	241.5462
-644.932	138.712	41.405	34.194	63.112	-83.85	333.1317
-646.324	157.924	41.405	32.369	84.15	-15.93	236.8784
Summary						
Valid Geometries		82				

Minimum Energy Value	-646.326

*The rows designated by 'ignorning geometries' refers to the geometries of first solvation shell from molecular simulations which are not able to converge due to poor repersenation of solvation shell surrounding the molecule. In case of PZ, a total of 82 geometries converged, with a minimum energy of -646.326 hartrees.

Molecular Geometries of ESS clusters for various Piperazine species (*x*,*y*,*z* Cartesian Coordinates in Å).

Piperazine (PZ)

1	14.436801000	12.445319000	11.200365000
6	13.701667000	12.402752000	11.996441000
6	13.451001000	11.844386000	14.342504000
6	12.345521000	10.858112000	13.980855000
6	12.593730000	11.413792000	11.651703000
1	13.010262000	12.798860000	14.612159000
1	12.776373000	9.860958000	13.848817000
1	13.035032000	10.443174000	11.407581000
1	13.281732000	13.398630000	12.098855000
1	14.009096000	11.492490000	15.203248000
1	11.621961000	10.796846000	14.786658000
1	12.048470000	11.750938000	10.776946000
7	14.386986000	12.075983000	13.244801000
1	14.955720000	11.260439000	13.110878000
7	11.668737000	11.308433000	12.772225000
1	10.930926000	10.668676000	12.537294000
8	7.264649000	13.360265000	12.674861000
1	8.190963000	13.525580000	12.861638000
1	6.798000000	13.508984000	13.485987000
8	10.010451000	13.674910000	13.306176000
1	10.585967000	12.922305000	13.124981000
1	10.543258000	14.455821000	13.240880000
8	9.393287000	9.381990000	11.866322000
1	8.597279000	9.869406000	11.649289000
1	9.109502000	8.517478000	12.129576000
8	7.020737000	10.881018000	11.320049000
1	6.729606000	11.075799000	10.439715000
1	7.045247000	11.717690000	11.789076000
8	15.458372000	14.787857000	13.906454000
1	15.265810000	13.870233000	13.714703000
1	16.381856000	14.837876000	14.108961000

Protonated Piperazine

1	14.107271000	14.741269000	12.645002000
6	13.617029000	13.778370000	12.588084000
6	13.514367000	11.739598000	11.313397000
6	12.016099000	11.889871000	11.107369000
6	12.122765000	14.007245000	12.434482000
1	13.699129000	11.113177000	12.189995000
1	11.808747000	12.426456000	10.192565000
1	11.916966000	14.610280000	11.561745000
1	13.810870000	13.252921000	13.526743000
1	13.931132000	11.231738000	10.453678000
1	11.504808000	10.937745000	11.093584000
1	11.685804000	14.464437000	13.309442000
7	14.101642000	13.056715000	11.430426000
1	15.100716000	13.017794000	11.411320000
7	11.436414000	12.691619000	12.229751000
1	11.478657000	12.145621000	13.084150000
1	10.448649000	12.849402000	12.052523000
8	8.536733000	13.107876000	11.900526000
1	8.314474000	13.708279000	12.620407000
1	8.049989000	13.395174000	11.137175000
8	8.736796000	10.398411000	12.915509000
1	8.367389000	11.201121000	12.553359000
1	8.065537000	9.728774000	12.873758000
8	8.363718000	14.698841000	14.155606000
1	9.001059000	14.440877000	14.820748000
1	7.733165000	15.278300000	14.563300000
8	10.911718000	10.944826000	14.586797000
1	10.107345000	10.595814000	14.193396000
1	11.350021000	10.221168000	15.018088000
8	10.552768000	13.623306000	15.662040000
1	10.825141000	13.860636000	16.539704000
1	10.618081000	12.673338000	15.592331000

Diprotonated Piperazine

6	11.838698000	11.396256000	13.732897000
6	11.940618000	11.619427000	11.265783000
6	13.296244000	12.292444000	11.373785000
6	13.190472000	12.060859000	13.864128000
1	11.910759000	10.319348000	13.662688000
1	12.024124000	10.545246000	11.147804000
1	13.220115000	13.367826000	11.422993000
1	13.096314000	13.129821000	13.997019000
1	11.227579000	11.647989000	14.587959000
1	11.403390000	12.003620000	10.407430000
1	13.921052000	12.051648000	10.528437000
1	13.731724000	11.652276000	14.707170000
7	14.001254000	11.843753000	12.625577000

1	14.264825000	10.869561000	12.540969000
1	14.874640000	12.341864000	12.729184000
7	11.147514000	11.883815000	12.499549000
1	10.243590000	11.459594000	12.455766000
1	10.913744000	12.884058000	12.614556000
8	7.604342000	12.423375000	12.923902000
1	7.464042000	11.831961000	12.186229000
1	6.715093000	12.748108000	13.081631000
8	8.513449000	13.631470000	10.120894000
1	8.625477000	13.553828000	9.179687000
1	9.163734000	13.094481000	10.549871000
8	16.786918000	13.940653000	13.023203000
1	16.724441000	14.880772000	13.168229000
1	17.654977000	13.678755000	13.314339000
8	15.668377000	13.752466000	10.359648000
1	16.121546000	14.287507000	9.719341000
1	16.238061000	13.673386000	11.118320000
8	9.850512000	14.134335000	13.294483000
1	9.051367000	13.591180000	13.383213000
1	9.582010000	14.985319000	12.957315000

Piperazine Carbamate

6	14.971896000	14.087384000	11.424184000
6	13.667213000	14.030760000	12.214344000
1	14.743788000	14.146989000	10.363782000
1	15.529508000	14.981666000	11.688830000
7	15.827936000	12.927006000	11.630178000
6	15.093565000	11.689627000	11.401432000
1	13.888249000	14.133017000	13.282859000
1	13.015900000	14.844163000	11.938313000
7	12.985130000	12.779826000	11.958272000
1	16.177194000	12.935897000	12.570951000
6	13.790818000	11.599767000	12.191843000
1	14.870793000	11.625969000	10.340461000
1	15.738588000	10.851707000	11.650187000
6	11.599177000	12.717606000	12.072041000
1	14.022513000	11.502100000	13.258565000
1	13.231508000	10.725984000	11.900792000
8	10.981586000	13.792323000	12.141409000
8	11.078746000	11.578957000	12.082403000
8	10.678004000	9.386416000	10.159848000
1	11.009916000	10.025258000	10.787424000
1	9.776856000	9.652950000	10.021905000
8	8.394432000	11.440297000	11.041375000
1	8.060665000	12.326587000	10.931501000
1	9.299195000	11.569541000	11.330595000
8	8.263192000	14.412607000	11.299633000
1	9.212616000	14.297095000	11.346213000

1	8.014148000	14.463167000	12.216959000
8	8.928462000	11.032473000	14.015230000
1	9.778607000	11.106697000	13.582823000
1	8.326307000	10.946313000	13.283498000
8	8.934802000	14.028464000	14.250289000
1	9.711219000	14.060880000	13.693052000
1	8.788471000	13.095509000	14.384811000

Protonated Piperazine Carbamate

1	14.830499000	13.256320000	10.771892000
6	14.083107000	13.503385000	11.507227000
6	13.693750000	13.718377000	13.865429000
6	12.490706000	12.784533000	13.819353000
6	12.882581000	12.578519000	11.327605000
1	13.374399000	14.749742000	13.724144000
1	12.788216000	11.765846000	14.013436000
1	13.175927000	11.543518000	11.385972000
1	13.783883000	14.538297000	11.349961000
1	14.165630000	13.639425000	14.830056000
1	11.703808000	13.064597000	14.507886000
1	12.355179000	12.744194000	10.397147000
7	14.638094000	13.372061000	12.831656000
7	11.890749000	12.803136000	12.438559000
1	11.428410000	13.686173000	12.293732000
1	11.180559000	12.074950000	12.390452000
6	15.506275000	12.277375000	13.092381000
8	16.038436000	11.757449000	12.094119000
8	15.628328000	11.946767000	14.269717000
8	14.918072000	9.634928000	10.565049000
1	15.335271000	10.386270000	10.991002000
1	15.581112000	8.955220000	10.586496000
8	12.978775000	9.161905000	12.658444000
1	13.587306000	9.062626000	13.396094000
1	13.537018000	9.163533000	11.881161000
8	17.341864000	9.298867000	12.536515000
1	17.053699000	10.215385000	12.477716000
1	18.251350000	9.316060000	12.800220000
8	15.122489000	9.143571000	14.531875000
1	15.315595000	10.076045000	14.642435000
1	15.853977000	8.828473000	14.008388000
8	10.519287000	10.364820000	12.486724000
1	9.745355000	9.818375000	12.500978000
1	11.289294000	9.789258000	12.578180000

Piperazine dicarbamate

6	12.689179000	12.162924000	11.966989000
6	13.204979000	12.514889000	14.293909000
6	14.653191000	12.115719000	14.036985000

6	14.133500000	11.751536000	11.700441000
1	12.594728000	13.231478000	11.744701000
1	13.125915000	13.605187000	14.235738000
1	14.772529000	11.054180000	14.276494000
1	14.208581000	10.661266000	11.751969000
1	12.022685000	11.625351000	11.310963000
1	12.907512000	12.216823000	15.286960000
1	15.314767000	12.677717000	14.677302000
1	14.426620000	12.058737000	10.708615000
7	15.028312000	12.364522000	12.661289000
7	12.314185000	11.894758000	13.336597000
6	16.365288000	12.619828000	12.345183000
6	10.955809000	11.709724000	13.667155000
8	16.670995000	12.523517000	11.123954000
8	10.179724000	11.497891000	12.719279000
8	17.128145000	12.967131000	13.245550000
8	10.660197000	11.750859000	14.873465000
8	10.394474000	9.869981000	16.949193000
1	10.294928000	10.549791000	16.273307000
1	11.173629000	9.408336000	16.672376000
8	18.402165000	14.429287000	10.173640000
1	17.789737000	13.755151000	10.509761000
1	18.849744000	14.728101000	10.953618000
8	7.510047000	11.576915000	12.002095000
1	7.798407000	11.823985000	11.135142000
1	8.343480000	11.504270000	12.486189000
8	17.604673000	10.744022000	9.239010000
1	17.339030000	11.262286000	10.009523000
1	16.896421000	10.874872000	8.626610000
8	19.612089000	12.683913000	8.160224000
1	19.348359000	13.397471000	8.735425000
1	19.120977000	11.935583000	8.491351000

Simulation Details

The molecular dynamics (MD) simulations were isothermal-isobaric simulations (nPT) with periodic boundary conditions at 298K and 1 bar. The particle-mesh Ewald procedure was used to handle long-range electrostatic interactions. Temperature was controlled by Langevin dynamics, while pressure was controlled by weak coupling to an external bath. The time-step in the simulations was set to 0.002 ps. Solute molecule geometries were fully constrained during simulations. Solute coordinates were restrained with a harmonic potential. The restraining weight was set at 5 kcal/mol Å2. The nonbonded cutoff was set to 8 Å for all simulations. Bonds length involving hydrogen atoms were maintained by use of a SHAKE algorithm. The systems were equilibrated in nPT simulations for 800 ps before geometries were extracted. Cluster geometries were extracted from the MD trajectory every 2 ps. These clusters consisted of the solute and the 5 closest solvent molecules.

Water solvent was represented with the TIP3P force field1. The number of solvent molecules in simulations for a given solute was taken to be 100. The solutes geometries were obtained from HF/6-31+G (d) calculations in vacuum. Solute charges were calculated with the CM2 model2.

The force field Lennard-Jones parameters were drawn from force fields reported in the scientific literature. We selected the force fields that appeared to have the most detailed parameterization for a given solute. For many ionic species no special force field have been developed, and is these cases we drew on atomic parameters for neutral species. For atoms where no force field stood out in terms of parameterization we drew on the GAFF force field3. GAFF would be our recommended default when no solute specific force field is available. Below are given the Lennar d Jones parameters utilized in the present work.

Table S2: Force Field Parameters

Atoms	Sigma [Å]	Epsilon [Kcal/mol]	Reference
O (OH^-, H_3O^+)	3.104	0.152	TIP3P water[1]
N	3.202	0.17	GAFF[3]
C	3.349	0.1094	GAFF[3]
C (Aromatic)	3.349	0.086	GAFF[3]
S	3.73	0.4945	Kristof and Lizli[4]
H	2.435	0.0157	GAFF[3]

H (N)	2.435	0.0157	GAFF[3]
H (Aromatic)	2.561	0.015	GAFF[3]

References

(1) Jorgensen, W. L.; Chandrasekhar, J.; Madura, J. D.; Impey, R. W.; Klein, M. L. J. Chem. Phys. 1983, 79, 926.

(2) Li, J.; Zhu, T.; Cramer, C. J.; Truhlar, D. G. J. Phys. Chem. A 1998, 102, 1820.

(3) Wang, J.; Wolf, R. M.; Caldwell, J. W.; Kollman, P. A.; Case, D.A. J. Comput. Chem. 2004, 25, 1157.

(4) Kristof, T.; Lizli, J. J. Phys. Chem. B 1997, 101, 5480.

QM/PB Continuum Solvent Model

In the PB models, the solution is characterized as a charge distribution of the solute in a cavity mimicking the molecular shape which surrounded by a continuous dielectric representing the solvent. The solvent is polarized by the solute and the solvent polarization generates an electrostatic field called the reaction field. The electrostatic potential of the reaction field can be described by the Poisson-Boltzmann equation:

$$((r) \quad (r)) - (r)^2 sinh((r)) = -4\,(r) \qquad (1)$$

The (r) is the charge distribution of the solute calculated via semi empirical QM methods. The (r) is the electrostatic potential that is to be obtained. The (r) is the dielectric function describing the dielectric discontinuity and it usually only has two values: $_{out}$ for the region outside of the cavity and $_{in}$ for the space within the (molecular) cavity. The (r) is a modified Debye-Huckel parameter that reflects the salt concentration and temperature. When the charge density on the solute is low and the ionic strength is low, the term can be approximated via a linear term yielding the so-called linear Poisson-Boltzmann equation:

$$[(r) \quad (r)] - (r)^2(r) = -4\,(r) \qquad (2)$$

Even for the linearized PB equation, analytical solutions can only be obtained for systems defined by a simple dielectric boundary, e.g., a sphere. For a boundary as complex as that of a protein or DNA molecule, the finite difference approach (among others) substitutes for the analytical differential equation solution yielding a numerical solution of the PB equation.

The electrostatic potential of the reaction field generated by a fixed set of (r) will, in turn, polarize the solute charge distribution and generate a new '(r) (i.e. distort the gas phase wave function). Thus the gas phase solute Hamiltonian, H^0 is perturbed by a potential energy operator coming from the interaction between the solute and the reaction field:

$$H^{'} = H^0 + V_{int} \qquad (3)$$

$$V_{int} = \int_s ds \frac{\sigma(r')}{|r - r'|} \tag{4}$$

The equation 4 is an integral over all the virtual surface charges, and r' is the coordinates of the surface charges. The virtual surface charges (σ) are calculated from the converged electrostatic potential of the reaction field. And the full set of virtual surface charges should generate an electrostatic field which is identical to the reaction field. Thus, the perturbation (Vint) due to the reaction field could be accurately calculated by equation 4, where r is the coordinates of each solute atom and r' loops through all the virtual surface charges. After the QM SCF converges with the perturbed H', the perturbed wave function is used to obtain a new solute charge distribution '(r) using CM1 or CM2 methods. A new PB equation constructed with the updated '(r) which then needs to be solved self-consistently again. This iterative calculation of QM and PB defines the so-called self-consistent reaction field (SCRF) calculation. The solvation free energy of the solute is obtained via equation 5 after the SCRF converges.

$$\Delta G_{solv} = \frac{1}{2}\sum_i Z_i . V_{int} + \langle \Psi' | H_M + V_{int} | \Psi' \rangle - \langle \Psi^0 | H_M | \Psi^0 \rangle + G_{np} = \left[\frac{1}{2}\sum_i Z_i . V_{int} + \langle \Psi' | V_{int} | \Psi' \rangle \right] + \left[\langle \Psi' | H_M | \Psi' \rangle - \langle \Psi^0 | H_M | \Psi^0 \rangle \right] + G_{np} = G_{rf} + G_{wfd} + G_{np} \tag{5}$$

The G_{rf} is the electrostatic interaction of the solute charge distribution (including core and electrons) with the electrostatic potential generated by the reaction field. It is more efficient and accurate to calculate the G_{rf} using the virtual surface charge, (see equation 6), instead of using the electrostatic potential directly.

$$G_{rf} = \frac{1}{2} \int_v \rho(r) \int_s ds \frac{\sigma(r')}{|r - r'|} \tag{6}$$

G_{wfd}, the wave function distortion energy is generated by the change of the gas phase wave function by the presence of solvent. It is the difference of the heat of formation calculated from polarized Fock matrix itself (without the interaction with the surface charges) and the heat of formation obtained in vacuum. G_{np}, the non-polar energy, contains contributions from cavity formation and solvent-solute dispersion-repulsion interactions. It is generally made proportional to the molecular surface area, using two surface tension parameters a & b (equation 7).

$$G_{np} = a * S + b \tag{7}$$

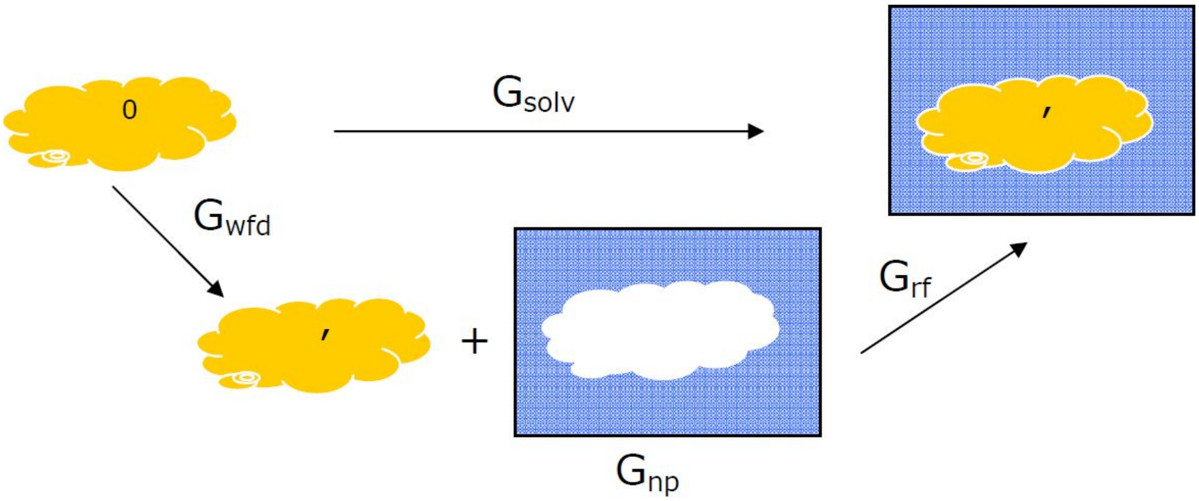

Figure 1 summarizes the origin of these three energy components of the solvation free energy

The components of the solvation free energy in the QM/PB model. G_{wfd} is the penalty originating from the polarization of the solute's wave function in vacuum relative to the wave function in solution. G_{np} models non-polar effects, including the entropy to make the molecular cavity in the solvent. G_{rf} is the electrostatic interaction between the polarized solute and the surrounding solvent.

Constant terms utilized in calculations

Dielectric constant of water: 78.39

The free energy of proton: -265.9 Kcal/mol.

$\Delta G_s(5H_2O)$ Poisson Boltzmann model: -15.7 kcal/mol

Energy for pure water clusters$(5H_2O)$: -380.133 [au]

Thermal energy correction and ZPE for pure water clusters$(5H_2O)$: 0.1435 [au]

Entropy for pure water clusters$(5H_2O)$: 114.8 [cal/mol]

Standard state correction utilized $(RTln[R^{'}T] - RTln(^{55.34}/5)$ = -3.32 kcal/mol

Full citation for References used in manuscript:

26. Y. Shao, L. F. M., Y. Jung, J. Kussmann, C. Ochsenfeld, S.T. Brown, A.T.B. Gilbert, L.V. Slipchenko, S.V. Levchenko, D.P. O'Neill, R.A. DiStasio Jr., R.C. Lochan, T. Wang,

G.J.O. Beran, N.A. Besley, J.M. Herbert, C.Y. Lin, T. Van Voorhis, S.H. Chien, A. Sodt, R.P. Steele, V.A. Rassolov, P.E. Maslen, P.P. Korambath, R.D. Adamson, B. Austin, J. Baker, E.F.C. Byrd, H. Dachsel, R.J. Doerksen, A. Dreuw, B.D. Dunietz, A.D. Dutoi, T.R. Furlani, S.R. Gwaltney, A. Heyden, S. Hirata, C-P. Hsu, G. Kedziora, R.Z. Khalliulin, P. Klunzinger, A.M. Lee, M.S. Lee, W.Z. Liang, I. Lotan, N. Nair, B. Peters, E.I. Proynov, P.A. Pieniazek, Y.M. Rhee, J. Ritchie, E. Rosta, C.D. Sherrill, A.C. Simmonett, J.E. Subotnik, H.L. Woodcock III, W. Zhang, A.T. Bell, A.K. Chakraborty, D.M. Chipman, F.J. Keil, A.Warshel, W.J. Hehre, H.F. Schaefer, J. Kong, A.I. Krylov, P.M.W. Gill and M. Head-Gordon. Spartan'08, Wavefunction, Inc. Irvine, Ca. *Phys. Chem. Chem. Phys.* **2006,** *8*, 3172-3191.

35. Wang, B.; Raha, K.; Liao, N.; Peters, M. B.; Kim, H.; Westerhoff, L. M.; Wollacott, A. M.; Van der Vaart, A.; Gogonea, V.; Suarez, D.; Dixon, S. L.; Vincent, J. J.; Brothers, E. N.; Merz K. M., J. **2007 DivCon**.

30. Higashi, M.; Marenich, A. V.; Olson, R. M.; Chamberlin, A. C.; Pu, J.; Kelly, C. P.; Thompson, J. P.; Xidos, J. D.; Li, J.; Zhu, T.; Hawkins, G. D.; Chuang, Y.-Y.; Fast, P. L.; Lynch, B. J.; Liotard, D. A.; Rinaldi, D.; Gao, J.; Cramer, C. J.; Truhlar, D. G. GAMESSPLUS, Version 2010-2. University of Minnestota; Minneapolis, MN, 2010, based on the General Atomic and Molecular Electronic Structure System (**GAMESS**) as described in Schmidt, M. W.; Baldridge, K. K.; Boatz, J. A.; Elbert, S. T.; Gordon, M. S.; Jensen, J. H.; Koseki, S.; Matsunaga, N.; Nguyen, K. A.; Su, S. J. General Atomic and Molecular Electronic Structure System. J. Comput. Chem. 1993, 14, 1347−1363.

42. D.A. Case; T.A. Darden; T.E. Cheatham, I.; C.L. Simmerling; J. Wang; R.E. Duke; R. Luo; R.C. Walker; W. Zhang; K.M. Merz; B. Roberts; S. Hayik; A. Roitberg; G. Seabra; J. Swails; A.W. Goetz; I. Kolossváry; K.F. Wong; F. Paesani; J. Vanicek; R.M. Wolf; J. Liu; X. Wu; S.R. Brozell; T. Steinbrecher; H. Gohlke; Q. Cai; X. Ye; J. Wang; M.-J. Hsieh; G. Cui; D.R. Roe; D.H. Mathews; M.G. Seetin; R. Salomon-Ferrer; C. Sagui; V. Babin; T.

Luchko; S. Gusarov; A. Kovalenko; Kollman, P. A. *University of California, San Francisco.* **2012 AMBER 12**.
